# Signal Mediators in the Implementation of Jasmonic Acid’s Protective Effect on Plants under Abiotic Stresses

**DOI:** 10.3390/plants12142631

**Published:** 2023-07-13

**Authors:** Yuriy E. Kolupaev, Tetiana O. Yastreb, Alexander P. Dmitriev

**Affiliations:** 1 Yuriev Plant Production Institute, National Academy of Agrarian Sciences of Ukraine, 61060 Kharkiv, Ukrainet_howk@ukr.net (T.O.Y.); 2 Educational and Scientific Institute of Agrotechnologies, Breeding and Ecology, Department of Plant Protection, Poltava State Agrarian University, 36003 Poltava, Ukraine; 3 Institute of Cell Biology and Genetic Engineering, National Academy of Sciences of Ukraine, 03143 Kyiv, Ukraine

**Keywords:** jasmonic acid, reactive oxygen species, calcium, gasotransmitters, antioxidant system, stomata, resistance to stress factors

## Abstract

Plant cells respond to stress by activating signaling and regulatory networks that include plant hormones and numerous mediators of non-hormonal nature. These include the universal intracellular messenger calcium, reactive oxygen species (ROS), gasotransmitters, small gaseous molecules synthesized by living organisms, and signal functions such as nitrogen monoxide (NO), hydrogen sulfide (H_2_S), carbon monoxide (CO), and others. This review focuses on the role of functional linkages of jasmonic acid and jasmonate signaling components with gasotransmitters and other signaling mediators, as well as some stress metabolites, in the regulation of plant adaptive responses to abiotic stressors. Data on the involvement of NO, H_2_S, and CO in the regulation of jasmonic acid formation in plant cells and its signal transduction were analyzed. The possible involvement of the protein components of jasmonate signaling in stress-protective gasotransmitter effects is discussed. Emphasis is placed on the significance of the functional interaction between jasmonic acid and signaling mediators in the regulation of the antioxidant system, stomatal apparatus, and other processes important for plant adaptation to abiotic stresses.

## 1. Introduction

Plant adaptation to stress factors is due to the functioning of a regulatory network consisting of hormonal and signaling mediators. Over the past three decades, it has been established that almost all classes of hormones are involved in plant adaptation: abscisic acid (ABA), cytokinins, auxins, gibberellins, salicylic acid, brassinosteroids, and jasmonates [1,2].

Jasmonic acid (JA) and its derivatives are one of the key groups of “stress” plant hormones [3,4,5]. A large body of experimental data has been accumulated showing the action of JA as a regulator of plant growth and development [6,7], as well as signal activating expression of plant defense genes during pathogen infestation [8,9] and plant damage by phytophages [10]. In recent decades, it has been demonstrated that JA also participates in the adaptive responses of plants to various abiotic factors such as extreme temperatures, drought, salinity, heavy metal ions, and ultraviolet radiation [3,9,11,12,13,14,15].

The stress-protective JA effects are realized through the participation of a cellular network of signaling mediators [16,17]. The role of the universal secondary messenger calcium is undeniable [18], although the mechanisms of its involvement in JA signaling have not yet been sufficiently investigated [19,20]. JA is known to have close functional links with reactive oxygen species (ROS) [17,21]. In the last decade, the functional interaction between JA and gasotransmitters has been intensively studied [12,16,22]. The term ‘gasotransmitters’ refers to small gaseous molecules synthesized by living organisms that perform signaling functions. Nitrogen monoxide (NO), carbon monoxide (CO), and hydrogen sulfide (H_2_S) are considered the main gasotransmitters in plants [23,24]. Gasotransmitters are known to be in close functional and, in some cases, direct chemical interaction with each other and with ROS [25,26,27]. Simultaneously, data on the involvement of these mediators in the physiological (stress-protective) effects of JA are still scattered and insufficiently analyzed. In particular, there are still no special reviews on the stress-protective effect of JA on plants concerning its functional interaction with all major gasotransmitters, NO, H_2_S, and CO. In recent years, data have been obtained on the functional links between JA and signaling mediators not only in traditional model organisms (Arabidopsis and tobacco) but also in several cultivated plants from different taxonomic groups.

This review attempts to analyze and summarize the data on the functional relationships of JA with calcium ions, ROS, and gasotransmitters as key elements of the plant cell signaling network and discusses the importance of these relationships for the protective effects of JA under abiotic stressors on plants of different taxonomic groups.

## 2. Stress-Induced JA Synthesis in Plants

**Figure 1:**
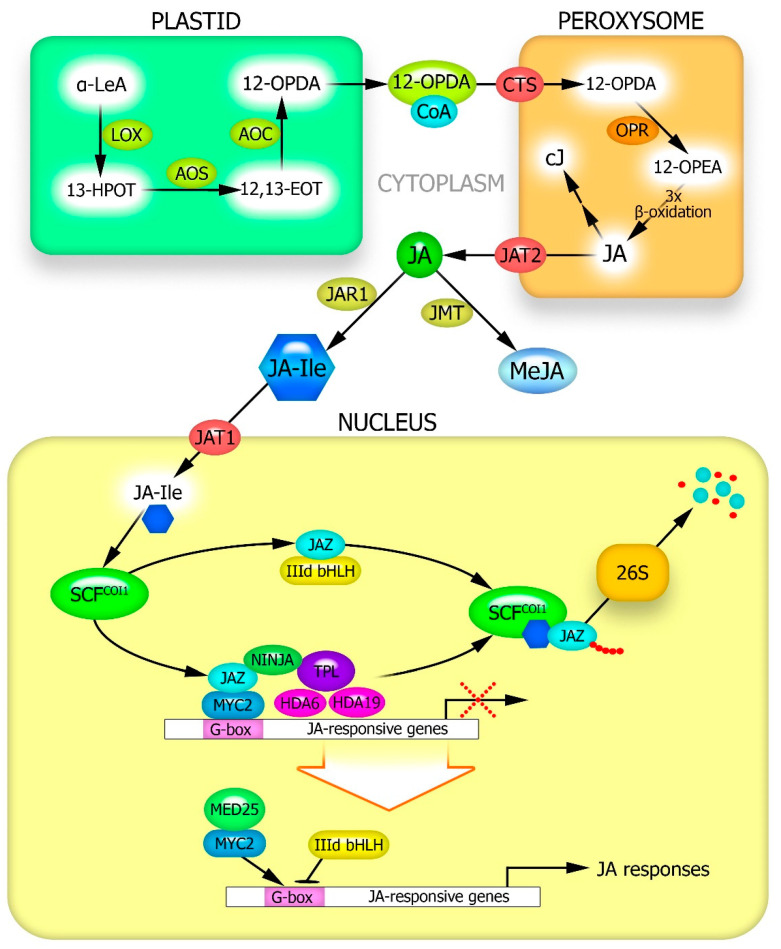
Main pathways of jasmonic acid synthesis and transduction of its signal. AOC—allene oxide cyclase; AOS—allene oxide synthase; cJ—*cis*-jasmone; COI1—CORONATINE INSENSITIVE1, F-box subunit of ubiquitin ligase complex; CTS—ABC transporter COMATOSE; 12,13-EOT—12,13-epoxy octadecatrienoic acid; HDA—histone deacetylase; 13-HPOT—13-hydroperoxy-linolenic acid; JA—jasmonic acid; JA-Ile—jasmonoyl-isoleucine; JAR1—jasmonate: amino acid synthetase 1; JAT—jasmonate transporter; JAZ—JASMONATE ZIM DOMAIN repressor protein; JMT—JA-carboxy methyl transferase; α-LeA—α-linolenic acid; LOX—lipoxygenase; MED25—MEDIATOR25 subunit of Mediator transcriptional coactivator complex; MeJA—methyl jasmonate; MYC2—bHLHzip transcription factor; NINJA—adaptor protein NOVEL INTERACTOR OF JAZ; 12-OPDA—12-oxo-phytodienoic acid; 12-OPEA—12-oxophytoenoic acid; OPR—12-OPDA reductase 3 (OPR3); SCF—Skp-Cullin-F-box complex; SCF^COI1^—ubiquitin ligase complex; TPL—TOPLESS co-repressor protein.

JJA formation in plants has been well studied and described in many reviews [3,5,28]. The process begins with phospholipase D cleaving linolenic acid (C18:3) from the lipid backbone of chloroplast membranes [29] (Figure 1). Linolenic acid is then oxidized to 13-hydroperoxy-linolenic acid by 13-lipoxygenase. The latter is dehydrated by allene oxide synthase, resulting in the formation of 12-oxo-phytodienoic acid (12-OPDA), which is delivered to peroxisomes via the ATP-binding cassette transporter protein CTS [30]. In peroxisomes, 12-OPDA reductase converts 12-OPDA into 12-oxophytoenoic acid. Three β-oxidation reactions of 12-oxophytoenoic acid produce JA [31] (Figure 1).

The main biologically active forms in plants are free JA, *cis*-jasmone, methyl jasmonate (MeJA), and jasmonoyl-isoleucine (JA-Ile) [32]. It has been suggested that the last two forms of jasmonate are formed after the transport of JA from peroxisomes into the cytoplasm by the AtJAT2 transporter [30]. Volatile MeJA is formed from JA through the activity of JA-carboxy methyl transferase [33]. Jasmonate: amino acid synthetase 1 (JAR1) catalyzes the reversible conversion of JA to JA-Ile [34]. *Cis*-jasmone is formed via JA decarboxylation in peroxisomes [30,32] (Figure 1).

It is well known that JA synthesis increases in response to infection by various pathogens and tissue damage caused by insects or mechanical impacts. However, JA accumulation in plants occurs under various abiotic stressors. For example, a transient increase in JA during drought has been recorded in Arabidopsis [32,35]. An increase in JA content in response to drought was also observed in tomato roots [36]. In wheat leaves, a five-fold increase in JA content has been recorded under drought conditions [9].

Under salt stress, an increase in JA has been shown in plant organs of Arabidopsis, tomato, potato, rice, and other species [32,37,38,39]. In barley under salt stress, activation of the expression of genes encoding enzymes involved in JA synthesis has been recorded: allene oxide synthase, lipoxygenase, and 12-OPDA reductase [40].

There is also evidence of increased JA synthesis in plants exposed to heavy metals. In Arabidopsis, cellular JA levels rapidly increase in response to treatment with Cd and Cu [41]. Exposure to 100 mM CuCl_2_ increased JA levels in rice 9-fold [42].

Cold-induced expression of lipoxygenase, allene oxide synthase, and allene oxide cyclase genes and increased JA content have been found in Arabidopsis and rice [43,44]. A similar effect in response to cold stress was found in annual wormwood plants [45].

JA synthesis can also be activated when plants are exposed to high temperatures. *Aquilaria sinensis* cell culture showed a significant transient increase in endogenous JA content after exposure to 50 °C [46]. Tobacco seedlings showed increased JA synthesis during several days of high-temperature (35 °C) treatment [47].

## 3. Proteins Involved in the Transduction of JA Signals into the Genome and Regulation of Jasmonate-Inducible Gene Expression

The greatest biological activity of JA is in the form of jasmonoyl-isoleucine, which is very low in the cytosol of plant cells under normal physiological conditions [32,34]. The conversion of JA to JA-Ile by JAR1 is induced by the action of stressors, with JA-Ile being transported to the nucleus (Figure 1). In *Arabidopsis thaliana*, this process is carried out by the transporter protein AtJAT1 [30,48]. JA-Ile is thought to be the only form of jasmonates capable of binding to the COI1 protein [49]. In the absence of abiotic stimuli, the JA-Ile content in cells is low, with the JAZ repressor proteins recruiting the TPL protein and the adaptor protein NINJA to form an active transcriptional repression complex, which inhibits the expression of jasmonate-responsive genes. The open complex is closed by further recruitment of the histone deacetylases HDA6 and HDA19 [30] (Figure 1). Abiotic stress increases JA synthesis, its conversion to JA-Ile and its transport to the nucleus. At a sufficiently high concentration, JA-Ile facilitates the interaction of JAZ with the COI1 protein within the SCF complex, leading to the proteasomal degradation of JAZ. Downstream transcription factors are activated to synergistically or antagonistically regulate JA-sensitive gene expression. The transcription factor MYC2 associates with the MED25 subunit of the Mediator complex binds to the G-box motif of the target promoter and activates JA-sensitive genes [28] (Figure 1). At the same time, factor IIId bHLH counteracts MYC2 via competitive binding to the G-box motif and inhibiting the expression of JA-sensitive genes.

Multiple JA-sensitive genes that are directly or indirectly controlled by MYC2 throughout JA signaling have been identified [50]. Moreover, bioinformatic analyses have shown that MYC2 can interact with at least 100 *A. thaliana* proteins [15]. Increased MYC2 expression has been reported in plants under stressors, particularly drought [30].

However, it should be noted that the JIN1/MYC2 signaling pathway does not appear to be the only pathway involved in JA signaling. For example, the jasmonate signal can be transmitted by ERF family proteins (ERF1, ERF2, ERF5, and ERF6), which combine the effects of JA and ethylene and are involved in regulating the expression of several genes [31,51]. There is also evidence that MYB family transcription factors are sensitive to jasmonate. Similar to MYC, these proteins can be directly repressed by JAZ proteins. The release of these transcription factors from JAZ, which occurs with an increase in cellular JA content, can activate their target genes [35]. Some transcription factors from the NAC family are sensitive to jasmonate. In Arabidopsis, the transcription factors of this family, AtAF1 and AtAF2, are induced by JA signals and are involved in adaptive responses to drought, salt, and oxidative stress [35,52]. JA can also affect transcription factors involved in the response of plants to low temperatures. Thus, treatment of rubber plants with exogenous MeJA eliminates the repressive effect of JAZ proteins on the transcription factor ICE2, which plays an important role in activating the C-repeat Binding Factor (CBF) cold signaling pathway involving *CBF1*, *CBF2*, and *COR47* genes. An increase in their expression was observed during the cold acclimatization of rubber trees [53].

## 4. Role of Calcium in JA Physiological Effects

Ca^2+^ is known to act as a universal secondary messenger involved in plant responses to stressors of various natures [54]. Thus, a significant increase in Ca^2+^ concentration in the cytosol of plant cells was observed in response to low [55] and high [56] temperatures, drought and salt stress [57], and other unfavorable abiotic factors. Calcium spikes are caused by the influx of Ca^2+^ ions through channels or their outflow by Ca^2+^-ATPase pumps and/or Ca^2+^/H^+^ ion exchangers [58]. The stress-induced increase in cytosolic calcium concentration is recognized, amplified, and transmitted to downstream signaling components by Ca^2+^-binding proteins. Calcium sensors in plants include calmodulin (CaM), calmodulin-like proteins (CML), calcineurin-B-like proteins (CBL), and Ca^2+^-dependent protein kinases (CDPK). CBL interacts with CBL-interacting protein kinases (CIPK) to form the CBL/CIPK signaling network, which plays a key role in plant responses to abiotic stress [54]. In this process, CBL activates CIPK, which phosphorylates CBL. Phosphorylation is the main mechanism that affects downstream proteins [59]. The specificity of calcium effects is thought to be due to differences in calcium concentrations and times of action on specific members of the calcium protein sensor network described above [54].

Calcium is involved in the formation of JA signaling and transduction. Along with other signaling mediators, it is also involved in the regulation of JA biosynthesis. Thus, the opening of calcium channels and Ca^2+^ entry into the cytosol are required for JA synthesis. In potato plants, the Ca^2+^ channel blocker ruthenium red inhibits the expression of JA biosynthesis genes [60]. It was later found that JA synthesis is regulated by calcium-dependent protein kinases [30]. For example, cold stress has been shown to increase cytosolic calcium levels in Arabidopsis cells, leading to an activation of gene expression of JA synthesis enzymes and JA signaling proteins [61]. In Arabidopsis, the calmodulin-like protein CML42 was also found to play an important role in calcium-mediated JA biosynthesis [62]. Recent studies have shown the role of several CMLs in transmitting drought signals and activating jasmonate signaling in response to this factor [63]. In addition to CMLs, the signaling chains leading to calcium-dependent activation of JA synthesis may involve extracellular ATP, catalytic complexes generating ROS (see below), and MAP kinase cascades [64].

In recent years, significant progress has been made in elucidating the mechanism by which calcium acts as a signaling mediator of JA synthesis in response to mechanical damage in plants. For example, a new JAV1-JAZ8-WRKY51 (JJW) complex was found in *A. thaliana*, which controls JA biosynthesis during defense against insect attacks. In intact plants, the JJW complex suppressed JA biosynthesis. At the same time, mechanical damage rapidly triggers a calcium influx to activate calmodulin-dependent phosphorylation of JAV1, which degrades the JJW complex and activates JA biosynthesis, causing a rapid burst of JA for plant defense [65]. In general, however, there are still insufficient data on the role of calcium and its connections with other signaling network components in JA synthesis regulation.

Several studies have shown the involvement of Ca^2+^ ions in JA signaling. Thus, in *A. thaliana*, the role of cyclic nucleotide-regulated channel 2 (AtCNGC2) in the jasmonic acid-induced entry of calcium into the cytosol from the apoplast has been established [19]. JA activates adenylate cyclase via an unknown pathway, resulting in an increase in [cAMP]_cyt_. cAMP, an activating ligand, causes the AtCNGC2 channel to open, leading to Ca^2+^ influx from the apoplast into the cytosol. In turn, this effect induces Ca^2+^ mobilization from intracellular calcium depots, such as the endoplasmic reticulum and mitochondria, leading to a further increase in [Ca^2+^]_cyt_ and the formation of strong calcium signals. Ca^2+^/CaM signaling modulates the activity of numerous and diverse CaM-binding proteins [19]. This signaling effect is reversible owing to calcium binding to CaM and the consequent inhibition of AtCNGC2.

The stress-protective effect of exogenous JA seems, in many cases, to be realized with the participation of calcium entering the cytosol from different pools. Thus, the increase in heat resistance of wheat coleoptile cells caused by exogenous JA was almost completely eliminated by both the calcium channel blocker lanthanum chloride and the extracellular calcium chelator EGTA [66]. It is possible that the physiological effects of JA are realized through the joint participation of calcium and ROS (see below). Exogenous JA increases NADPH oxidase-dependent superoxide anion radicals and hydrogen peroxide generation. In turn, the effect of NADPH oxidase activation was eliminated by calcium antagonists [66]. NADPH oxidase is known to be both directly and indirectly activated by calcium ions [67,68]. Both ROS and calcium appear to be involved in JA-induced activation of stress-protective systems, including antioxidant systems, and as a result, increase cellular heat resistance [21]. The mitigation of arsenic toxicity in tomato plants by JA treatment was also shown to be calcium-dependent, as it was eliminated by EGTA [69].

In the stomatal response of plants to drought, there is a functional interaction between JA and ABA. The intracellular Ca^2+^ content is regulated to a much greater extent by JA than by ABA [20]. The calcium channel blockers ruthenium red and lanthanum chloride, as well as the calmodulin antagonist trifluoperazine, completely reversed the stomatal closure effect of methyl jasmonate treatment in Arabidopsis [70]. At the same time, treatment with the extracellular calcium chelator EGTA only partially relieved the stomatal closure effect caused by MeJA, indicating a special role of calcium entry into the cytosol from intracellular depots and its binding to calmodulin for the realization of JA stomatal effects. More recent work has also confirmed that stomatal closure induced by JA is due to the activation of CDPK-dependent signaling pathways [71]. Bioinformatics and transcriptomics studies have shown that CaM regulates various transcription factors called CAMTA [20]. CAMTAs include the bZIP, MYB, WRKY, and NAC families of transcription factors that control many plant defense responses under stress conditions [72,73].

## 5. Involvement of ROS and Antioxidants in Stress-Protective Action of JA

ROS include mutually convertible reactive oxygen species, most of which exist for a short time. These include free radical particles such as superoxide anion radical (O_2_^•−^), hydroxyl (^•^OH), hydroperoxyl (HO_2_^▪^) radicals, etc., as well as neutral molecules like hydrogen peroxide (H_2_O_2_), organic peroxides (ROOH), singlet oxygen (^1^O_2_), etc. [74,75].

In plant cells, chloroplasts, mitochondria, peroxisomes, plasma membranes, and cell walls are known to be the main compartments of ROS formation [75,76]. The physiological effects of JA have been shown to involve ROS produced by the plasmalemma and cell wall enzymes [77,78].

The plasma membrane is an important center of ROS production owing to the presence of bound NADPH oxidase, also known as the respiratory burst oxidase homologue (RBOH). RBOH can transfer free electrons from its intracellular region to molecular oxygen on its outer surface [79]. The RBOH complex protein consists of the membrane-bound catalytic subunit RBOH, its cytosolic regulator ROP (Rho-like protein) and an integral plasmalemma protein consisting of six transmembrane domains connected by five loops. The TMD-3 and TMD-5 domains contain a pair of His residues that are required for binding to the heme group. The enzyme also contains hydrophilic FAD and NADPH domains and two N-terminal Ca^2+^-binding EF-hand motifs, which enable the regulation of RBOH activity by calcium ions [76].

Cell walls are also an important compartment for ROS accumulation through the activity of enzymes such as class III peroxidase, amine oxidase, and oxalate oxidase [80]. Thus, cell wall peroxidase, together with NADPH-oxidase of the plasma membrane, can oxidize NADPH and stimulate O_2_^•–^ production, which is considered a component of the signals necessary for the development of adaptive plant responses to abiotic stress conditions [81]. ROS are involved in realizing the signaling potential of other signaling mediators, particularly calcium ions [76] and gasotransmitters [82], as well as many plant hormones including JA. One of the results of this interaction of signaling mediators under biotic and abiotic stress is the activation of Ca^2+^-dependent protein kinases and their modulation of the state of target transcription factors and gene expression [83].

Changes in ROS levels may induce JA synthesis. For example, it has long been shown that ROS mediates the transient enhancement of JA synthesis in *Taxus chinensis* cell suspension culture induced by ultrasound treatment [84]. The inhibition of ROS accumulation by exogenous ascorbic acid, superoxide dismutase (SOD), catalase (CAT), or the NADPH oxidase inhibitor diphenyleneiodonium eliminated the effects of increased lipoxygenase and allene oxide synthase activity and JA accumulation in cells caused by the action of ultrasound. In addition, based on the experimental data obtained from various objects, a model for the induction of JA accumulation in plants under mechanical stress (wounding) was proposed. The components of this effect are the opening of mechanosensitive calcium channels, subsequent activation by calcium of the catalytic subunit of NADPH oxidase, increased generation of ROS as well as extracellular ATP, activation of MAP kinases and, consequently, increased expression of JA synthesis genes [64].

Hu et al. [85] proposed a model explaining the involvement of ROS in both the effects of JA and the induction of its synthesis. Thus, the treatment of tomato leaves with exogenous JA caused a significant increase in plasma membrane NADPH-oxidase activity and ROS accumulation; these effects were blocked by pretreatment with the NADPH-oxidase inhibitor diphenyleneiodonium. In contrast, the addition of diphenyleneiodonium to ginseng cell culture blocked pathogen elicitor-induced JA synthesis. The authors suggested that ROS generation is necessary as a signal for elicitor-induced JA synthesis in plants under biotic stress. At the same time, the synthesized JA causes secondary ROS accumulation that is more intense and directly necessary for the induction of protective gene expression [85].

Combined exposure of carrot roots to UV radiation and wounding caused a rapid increase in ROS formation, which acted as a signal for ethylene biosynthesis, in turn activating JA biosynthesis [86]. At the same time, the wound-induced increase in the expression of the 12-oxophytodienoate-10,11-reductase (OPR) gene, a key enzyme in JA biosynthesis, was not inhibited by the NADPH oxidase inhibitor diphenyleneiodonium or the lipoxygenase inhibitor phenidone. However, it was leveled when the two inhibitors were used together, which, according to the authors, indicated the joint involvement of ROS and endogenous JA in the regulation of OPR gene expression under wound stress [87]. In general, however, data on ROS participation in stress-induced JA synthesis, especially under the action of abiotic factors in plants, are still insufficient, and the specific role of ROS in the induction of JA synthesis enzyme gene expression cannot be unequivocally proven.

However, a number of studies have shown an increase in ROS formation in plant cells under JA action. On Arabidopsis plants not only the role of ROS in the physiological effects of JA was shown, but also data on the significance of specific proteins of jasmonate signaling for ROS formation under the action of exogenous JA were obtained [88]. In particular, based on the information about the absence of an increase in ROS generation under JA action in *coi1* mutant plants, it was concluded that jasmonate signaling protein COI1 is involved in the effect of JA on redox metabolism [88]. In contrast, the H_2_O_2_ content of *myc2* mutants during methyl jasmonate treatment did not differ from that of the wild-type plants. The authors concluded that COI1-dependent ROS production by AtRbohs occurs upstream of MYC2 during JA signaling [88].

It is likely that ROS, as mediators, are also involved in JA induction of plant defense responses to abiotic stresses. As mentioned above, the increase in heat resistance of wheat coleoptiles by exogenous JA was accompanied by a calcium-dependent increase in ROS generation [66]. The ROS scavenger ionol, NADPH-oxidase inhibitor imidazole, and peroxidase inhibitor salicylhydroxamic acid eliminated the JA-induced effect of increased superoxide anion radical formation by the coleoptile cell surface and the subsequent increase in their resistance to heating. In this case, at least two enzymes are thought to be involved in the formation of ROS signaling pool: NADPH oxidase and cell wall peroxidase [66]. As already noted, plant extracellular peroxidases have the ability to generate O_2_^•−^; it is particularly evident in the presence of excess reducing agents [81]. In addition to NADPH oxidase and extracellular peroxidase, polyamine oxidase may be involved in JA-induced ROS formation in the apoplast. This information was obtained in maize (*Zea mays*) plants exposed to exogenous methyl jasmonate [89].

While there are many data indicating an increase in the activity of ROS-generating enzymes (especially NADPH oxidase), there are also data on the suppression of the activity of this enzyme by JA. In rice plants under cyanide-induced oxidative stress, exogenous JA reduces ROS content, NADPH oxidase activity, and expression of individual genes encoding the catalytic subunit of the enzyme [90]. The molecular mechanisms underlying the effects of JA on ROS-generating systems remain poorly understood. Based on the available phenomenological data, we can assume that such effects of JA may be determined by other signaling mediators, particularly calcium ions, as mentioned above. It is not excluded that under different conditions, various mediators may be involved in the modulation of ROS generation systems by jasmonic acid, which may lead to effects that differ not only in strength but also in sign. Also, other hormones, especially salicylic acid, ABA, and ethylene, may be involved in the modulation of ROS generation under JA action, which themselves cause signals that alter redox homeostasis and are in complex functional relationships with JA [91]. A discussion on these connections is beyond the scope of this review.

JA signaling not only regulates ROS generation but also activates the antioxidant system, preventing the development of oxidative stress, which is a satellite of stress influences of various natures [21]. In wheat plants, not only ROS but also MEK1/2 protein kinase has been shown to be involved in the jasmonate-induced increase in the activity of ascorbate-glutathione cycle enzymes [92]. In this case, ROS levels are higher in the signaling cascade that induces antioxidant enzymes than MEK1/2 and causes phosphorylation of this protein, which is necessary for further activation of the antioxidant system.

An increase in the activity of antioxidant enzymes under the influence of JA has also been reported in many other plant species. Thus, treatment of barley plants with JA increased salt tolerance, which, according to the authors, was largely due to an increase in SOD, CAT, and peroxidase (POD) activity [93]. Under the influence of seed pretreatment and soybean plants spraying with jasmonic acid, gene expression and activity of Fe-SOD, non-specific peroxidase, catalase, and ascorbate peroxidase (APX) in leaves and roots under saline conditions were significantly increased, and oxidative stress was mitigated [94]. Additionally, an increase in sugars and phenolic compounds with antioxidant properties in soybean leaves under salt stress has been reported under the influence of JA spraying [95].

Indices of antioxidant system functioning (activity of SOD, catalase, non-specific peroxidase, amount of ascorbate, reduced glutathione, and phenolic compounds) increased in maize plants sprayed with JA when exposed to lead [96]. The effect of enhancing resistance to the nickel toxic effect by treatment with JA in soybean plants was accompanied by an increase in activity and expression of the SOD, CAT, POD, and APX genes [97]. Enhanced gene expression and increased activities of SOD, ascorbate peroxidase, catalase, glutathione reductase, and ascorbate and GSH contents have been found in leaves of JA-treated Brassica napus plants exposed to arsenic toxicity [98]. The role of JA in the regulation of the ascorbate-glutathione cycle in apples (*Malus pumila*) under cold stress has been demonstrated [99]. Exogenous JA stimulates ascorbate accumulation in cells mainly by increasing dehydroascorbate reductase activity. In contrast, treatment of plants with the JA synthesis inhibitor ibuprofen significantly reduced the activity of antioxidant enzymes (ascorbate peroxidase, dehydroascorbate reductase, and monodehydroascorbate reductase), indicating its involvement in the regulation of the antioxidant system.

To study the involvement of the antioxidant system in the realization of JA protective action under abiotic stresses, a number of studies have used mutants defective in JA signal transduction genes or JA synthesis genes. Thus, it was shown that the activities of SOD, catalase, and guaiacol peroxidase in Arabidopsis *jin1* leaves under salt stress (150 mM NaCl) were lower than those in wild-type plants [100]. At the same time, in *coi1* mutants, only the activity of guaiacol peroxidase was reduced, whereas the activity of the other two enzymes under salt stress did not differ from the values characteristic of wild-type plants. However, exogenous methyl jasmonate caused an increase in the activity of antioxidant enzymes in wild-type plants but did not affect their indices in either mutant.

Jasmonate-deficient *def-1* tomato plants showed more pronounced symptoms of oxidative stress when grown under saline conditions than wild-type plants. The reduced salt tolerance of *def-1* plants is associated with lower antioxidant enzyme activity and lower non-enzymatic antioxidants [101]. A different picture was observed when comparing the salt stress response of the antioxidant system in maize plants of the wild-type and JA synthesis mutants (*opr7opr8*). Jasmonate-deficient mutants accumulated less ROS in their leaves under salt stress than wild-type plants. They showed higher activity of catalase and ascorbate peroxidase under salt stress but lower activity of guaiacol peroxidase than wild-type plants. Glutathione reductase and glutathione-S-transferase activities in the leaves of jasmonate-deficient mutants under salt stress were also higher than those in wild-type plants. These indicators were lower in the roots of *opr7opr8* plants [102]. Thus, the reduced content of JA in certain plant species may increase their resistance to oxidative stress, although significantly more examples show positive regulation of the antioxidant system state by jasmonates. In general, the effects of gene expression activation and antioxidant enzyme activity enhancement involving JA have been detected in different plant species and under stress factors of various natures [3].

Thus, available evidence suggests that JA synthesis may be induced by enhanced ROS formation in cells under the influence of stressors [86], and JA is able to activate various components of the antioxidant system, probably through enhanced production of ROS as signaling mediators. At the same time, there is recent evidence suggesting that JA synthesis may be regulated by compounds classified as antioxidants. Zhang et al. [103] showed that Arabidopsis plants transformed with the flavonoid synthetase genes *AvF3′H*, *AvF3H*, and *AvFLS* from the medicinal plant *Apocynum venetum*, characterized by their high flavonoid content, accumulated significant amounts of JA in response to salt stress. The authors also documented an increased expression of genes encoding proteins involved in JA synthesis and signal transduction. No such effects were observed in wild-type plants under salt stress. Transformants with a higher flavonoid content and JA content exhibited higher tolerance to salinity and oxidative stress. Recently, the ability of proline, which also has antioxidant properties [104], to induce JA synthesis under cadmium toxicity has been shown in rice plants [105]. Treatment with proline, through activation of the jasmonate signaling pathway, which includes MYB transcription factors, inhibits the synthesis of anthocyanins, which in excessive accumulation become agents of oxidative stress [105].

## 6. The Connection between Jasmonate Signaling and Gasotransmitters

### 6.1. Nitric Oxide (NO)

Nitric oxide is the most studied gasotransmitter in animal and plant cells [106,107]. NO in plants can be produced via reductive or oxidative pathways [108,109]. NO synthesis by reduction can use nitrate or nitrite as substrates in reactions catalyzed by nitrate reductase, plasma membrane-bound nitrite: NO reductase and peroxisome-localized xanthine oxidoreductase [110,111].

The mechanism of NO formation in plants by the oxidative pathway from L-arginine is still a matter of debate, as animal NO synthase (NOS) homologs have only been found in green algae and not in higher plants [112]. Currently, terrestrial plants are considered to have no typical animal NOS. It is thought that this gene was lost during evolution [113]. However, several studies on corn, peas, and tobacco have shown that the use of NOS inhibitors and monoclonal or polyclonal antibodies reduced NO production in plants. In this regard, the existence of NOS-like enzymes in plants is assumed, but their genes and amino acid sequences are significantly different from those in animals [114]. In particular, several maize NOS-like proteins have been found to exhibit restricted sequences that are homologous to animal NOS sequences. As early as 20 years ago, a protein produced by the *AtNOS1* gene was identified in Arabidopsis, with a sequence similar to that of the protein involved in NO formation in snails [115]. Later it was found that AtNOS1 is a cGTPase, which was renamed AtNOA1 [116]. It is now thought that this protein may indirectly regulate NO formation [114].

Although NO has long been regarded as a signaling mediator in plant cells, the mechanisms of its signal transduction into the genetic apparatus are yet to be fully elucidated. This signal transduction may involve ROS, cGMP, calcium ions, and other mediators [117,118,119,120]. A very important role in the physiological effects of NO is its ability to induce post-translational modifications of proteins, nitrosation of thiol groups, and nitration by tyrosine. In general, these processes are important in regulating the activity of pro- and antioxidant enzymes and cellular redox homeostasis [27]. The effect of NO on ROS depends on the local concentrations of nitric oxide and its interactions with other mediators and can vary significantly with time. As already noted, JA can trigger the enhancement of both ROS and nitric oxide formation, and their location in signaling circuits is extremely difficult to determine experimentally. It is not uncommon to observe a simultaneous and interdependent increase in both mediators under the influence of JA [121].

**Figure 2:**
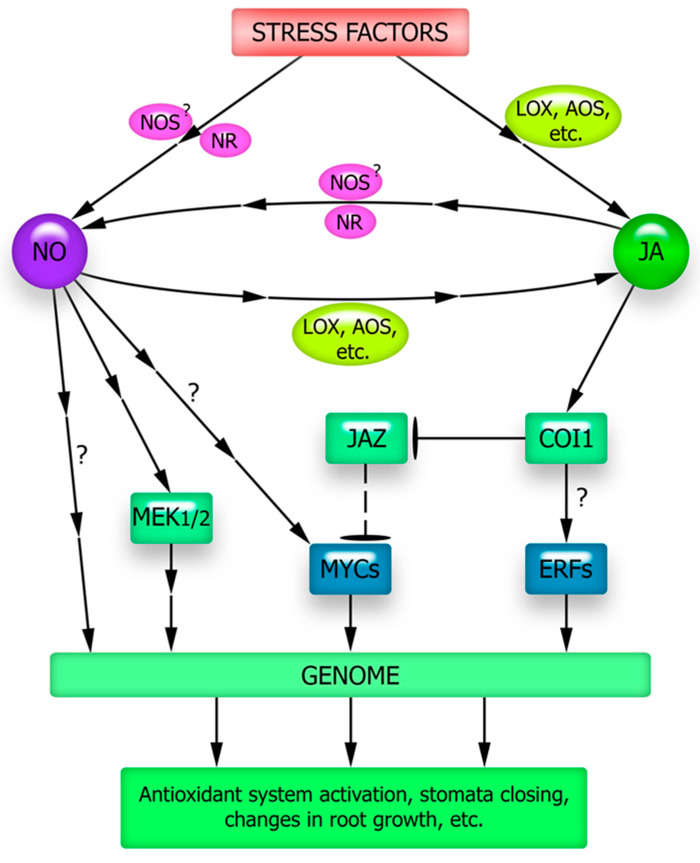
Possible ways of functional interaction of JA and NO in the formation of plant adaptive responses. AOS—allene oxide synthase; COI1—CORONATINE INSENSITIVE1 (ubiquitin ligase complex involved in protein degradation in the 26S proteasome); JAZ—JASMONATE ZIM DOMAIN repressor protein; LOX—lipoxygenase; MEK1/2—protein kinase MEK1/2; MYCs—transcriptional factors of the MYC family; NOS—NO synthase; NR—nitrate reductase. See the explanations in the text.

There seems to be a considerable intersection in the signaling pathways of nitric oxide and JA as plant stress hormones (Figure 2). Several experimental data suggest a role for NO in JA synthesis [122]. Almost two decades ago, it was shown that in Arabidopsis plants there is a rapid increase in nitric oxide generation in response to wounding, which in turn causes an increase in the activity of JA synthesis enzymes, lipoxygenases and allene oxide synthase, and an increase in JA content in leaves [123]. Enhanced expression of key genes involved in JA synthesis under the influence of exogenous NO has also been shown [124,125,126]. UV-B irradiation of *Panax quinquefolius* increased the formation of nitric oxide, jasmonic acid, and triterpene glycoside ginsenosides in the roots. The UV-B-induced increase in ginsenoside content was eliminated by the NO scavenger PTIO (2-phenyl-4,4,5,5-tetramethylimidazoline-1-oxyl-3-oxide), NO synthase inhibitor L-NAME (N^G^-nitro-L-arginine methyl ester), and JA synthesis inhibitor salicylhydroxamic acid. Treatment with NO antagonists inhibited UV-B-induced JA accumulation, suggesting that NO is located upstream of the JA signaling pathway [127].

In *Sophora flavescens*, NO treatment caused an increase in lipoxygenase activity and JA levels. In turn, exogenous JA application stimulates NO production associated with L-arginine oxidation [128]. Thus, a possible reciprocal enhancement of JA and NO synthesis in S. flavescens cells was demonstrated. In contrast, exogenous NO was found to reduce JA accumulation caused by As(III) toxicity in rice plants [129]. According to the authors, this effect is due to the NO donor mitigating the toxic effects of As on the plants. However, it is possible that NO may be involved in both the direct and reverse regulation of JA content in plant tissues.

One mechanism of the functional interaction between JA and NO may be related to the involvement of the same regulatory transcription factors in realizing their effect on gene expression. The transcription factor JIN1/MYC2 is considered a transduction node for many stress-related signals in plant cells [130]. Based on bioinformatics analysis, it has been suggested that *MYC* family genes are involved in the signal transduction of NO [131]. Experimental evidence also points to the possibility of such an involvement. Thus, under salt stress, Arabidopsis *jin1* mutants defective in the JIN1/MYC2 protein gene were weakly sensitive to the protective effect of the nitric oxide donor sodium nitroprusside (SNP). At the same time, treatment of wild-type plants with this NO donor increased the activity of the antioxidant enzymes SOD, catalase, and peroxidase, as well as the sugar content and mitigated the oxidative damage caused by salt stress [132]. Naturally, it cannot be excluded that the effects of exogenous nitric oxide on the protective systems of Arabidopsis plants can also be mediated by its effect on JA content in the signaling of which JIN1/MYC2 is a key protein. The absence of the effect of NO on the antioxidant system functioning of *jin1* mutants under salt stress suggests that NO-dependent signals required for salt tolerance formation may be implemented with the participation of the JIN1/MYC2 transcription factor [132] (Figure 2).

Data indicating the involvement of jasmonate signaling components in the physiological effects of nitric oxide were also obtained when studying the effect of nitric oxide donor SNP on lateral root growth in Arabidopsis plants. SNP treatment suppressed root growth and increased lateral root density in wild-type plants, but this response was significantly reduced in *coi1-1* mutants. The authors concluded that the JA receptor COI1 is involved in the effects of JA and nitric oxide [16].

The data available in the literature not only indicate the influence of nitric oxide on JA synthesis and the involvement of components of jasmonate signaling in realizing its effects but also indicate the involvement of NO in the transduction of JA signals into the genetic apparatus (Figure 2). For example, JA and NO have been shown to be involved in the control of allantoin synthesis in sugar beet plants, which is important for their adaptation to saline-alkaline soil [133]. Inhibition of JA biosynthesis completely eliminated the resistance of plants to alkaline salinity induced by exogenous allantoin and the accumulation of NO. In addition, there was no NO donor-induced increase in salt tolerance upon the suppression of JA synthesis. In contrast, inhibition of NO biosynthesis attenuates allantoin-induced tolerance to alkaline salinity, JA accumulation, and JA-induced plant tolerance to saline-alkaline stress [133]. These data experimentally supported the assumption of different levels of functional interactions between JA and NO. They manifest themselves in the influence of JA and NO on each other’s synthesis (the presence of a self-reinforcing feedback loop) and in the involvement of signal transduction (Figure 2).

Treatment of cucumber fruit with methyl jasmonate and the nitric oxide donor SNP reduced oxidative damage during low-temperature storage [134]. This effect was accompanied by an increase in CAT1 and CAT3 gene expression as well as overall catalase activity, resulting in a decrease in hydrogen peroxide content in the fruit. At the same time, the endogenous NO accumulation inhibitors, L-NAME and PTIO, eliminated the development of cold tolerance induced by methyl jasmonate. In contrast, the JA synthesis inhibitors ibuprofen and salicylhydroxamic acid had no effect on the SNP-activated cold tolerance of the fruits. The authors, therefore, suggested that NO mediates the methyl jasmonate signaling pathway, which activates cold tolerance in cucumbers [134].

The activation of ascorbate and glutathione metabolism enzymes by exogenous JA in maize plants was mediated by an increase in NO synthesis required for the phosphorylation of MEK1/2 protein kinase, which, in turn, was necessary to increase APX, GR, MDHAR, and DHAR activity [135] (Figure 2).

A synergistic effect was observed in a study on the protective effect of JA and NO on tomato plants under salt stress [136]. The authors showed that combined treatment of plants with the NO donor S-nitroso-N-acetyl penicillamine and JA had a more significant positive effect on plant growth, photosynthetic pigments, low-molecular-weight antioxidants (flavonoids, ascorbate, and GSH), and antioxidant enzyme activity (SOD and catalase) compared to the effect of each agent alone. In addition, the combination of S-nitroso-N-acetyl penicillamine and JA was more effective in mitigating the effects of salinity-induced oxidative stress [136].

The ability of JA and some other oxylipins to induce stomatal closure is known [137,138]. The effect of JA on stomatal status is mediated by major cellular signaling mediators, particularly ROS and nitric oxide. An increase in H_2_O_2_ and NO was observed in the guard cells of *A. thaliana* and *Vicia faba* under the action of JA or MeJA [139,140]. It is thought that the enhancement of ROS generation during induction of JA stomatal reactions is mainly due to an increase in NADPH oxidase activity [141], while the increase in NO is predominantly due to nitrate reductase involvement [142]. Furthermore, a mathematical model is proposed in which the functional interaction of NO, ROS, and antioxidants integrates the regulation of stomata by the three main plant hormones controlling this process, ABA, methyl jasmonate, and ethylene [142].

However, the mechanisms underlying increased NO synthesis in guard cells under the influence of JA and methyl jasmonate have not been fully elucidated. In the bean leaf epidermis, stomatal closure induced by JA is eliminated by the NO synthase inhibitor L-NAME [139]. The MeJA-induced reduction in stomatal gap size and a number of open stomata in Arabidopsis was almost eliminated by treatment of epidermal cells with an NO PTIO scavenger and partially with inhibitors of animal NO synthase (L-NAME) and nitrate reductase (sodium tungstate) [143]. Thus, it is possible that stomatal closure under the action of methyl jasmonate is mediated by both oxidative and reductive pathways of NO synthesis. However, it has been shown in the epidermis of *Vicia faba* that inhibition of nitrate reductase does not reverse the stomatal closure effect caused by JA [139]. It is not excluded that the contribution of different pathways of JA-induced NO synthesis in guard cells may vary according to plant species and age as well as experimental conditions.

### 6.2. Hydrogen Sulfide

Hydrogen sulfide is now seen as a gasotransmitter involved in the regulation of many plant functions, in particular, growth processes, fruit ripening and aging, adaptation to stressors of various natures [144,145,146,147].

One of the main pathways for H_2_S synthesis in plants is the catalytic conversion of L-cysteine to pyruvate by L-cysteine desulfhydrase, which releases hydrogen sulfide and ammonium [148]. The signaling effects of hydrogen sulfide are largely related to protein persulfidation, that is, the conversion of the cysteine-thiol group (-SH) into the corresponding persulfide (-SSH) [149,150,151]. It is believed that H_2_S or its ionic forms (HS^−^ and S^2−^) cannot directly react with protein thiols. It is likely that H_2_S interacts with oxidized cysteine residues (R-SOH) [152]. In this case, the process of protein modification is triggered by ROS signaling and oxidation of the cysteine thiol group to sulfenic acid by hydrogen peroxide. Sulfenic acid residues interact with H_2_S to form persulfide groups [153].

Persulfidation appears to be one of the key tools for the regulation of gene expression. Activation of the expression of genes encoding many transcription factors and chromatin modifiers was observed when Arabidopsis plants were treated with exogenous H_2_S [154,155]. In tomatoes, during NaHS root treatment, 5349 genes were activated and 5536 genes were suppressed [156].

The effects of hydrogen sulfide can be seen not only in the direct regulation of the redox state of protein molecules but also in the induction of calcium and ROS signals [82,157]. On the other hand, hydrogen sulfide synthesis itself is also dependent on ROS and calcium homeostasis [135,158,159].

Hydrogen sulfide also has close functional interactions with nitrogen oxide. One mechanism for this interaction is related to the competition between two key gasotransmitters for thiol groups in protein post-translational modification processes [27]. Also, NO and H_2_S can affect each other’s synthesis [160,161,162,163].

**Figure 3:**
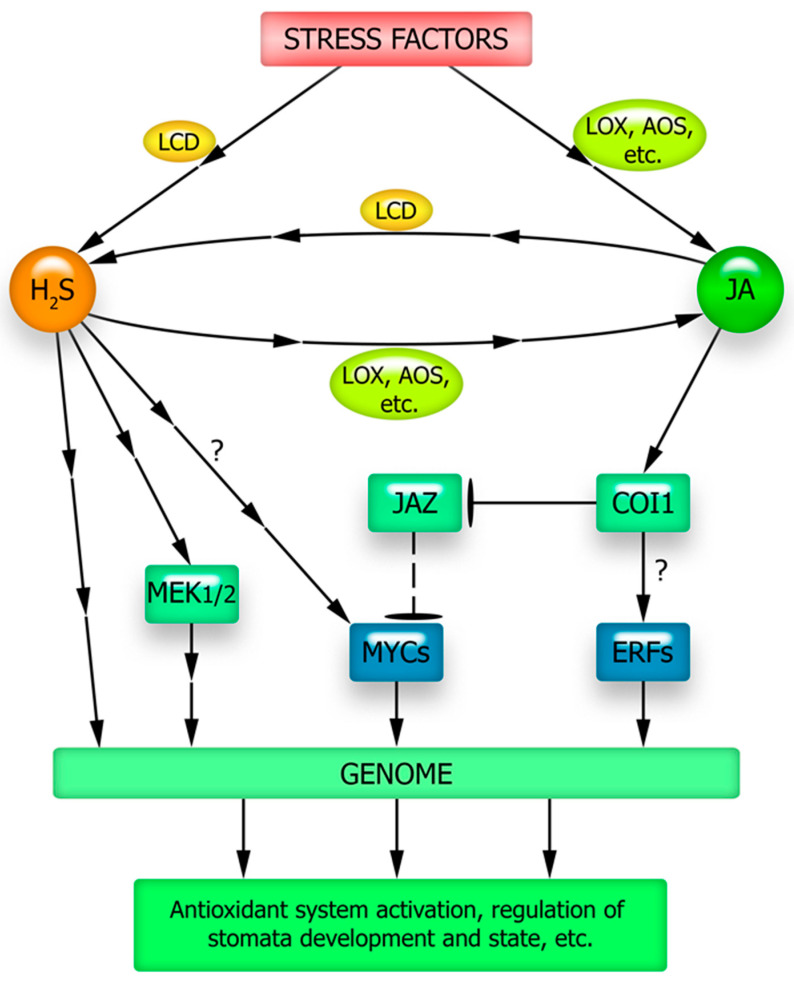
Possible ways of functional interaction of JA and H_2_S in the formation of plant adaptive responses. AOS—allene oxide synthase; COI1—CORONATINE INSENSITIVE1 (ubiquitin ligase complex involved in protein degradation in the 26S proteasome); JAZ—JASMONATE ZIM DOMAIN repressor protein; LCD—L-cysteine desulfhydrase; LOX—lipoxygenase; MEK1/2—protein kinase MEK1/2; MYCs—transcriptional factors of the MYC family. See the explanations in the text.

JA appears to be an important component of the hydrogen sulfide-activated signaling network (Figure 3). Hydrogen sulfide fumigation of foxtail millet plants has been shown to increase their methyl jasmonate content and induce resistance to the toxic effects of cadmium [164]. Treatment of tomatoes with H_2_S donors increased the JA content in leaves in the control and under salt stress [165].

The adaptation of tobacco plants to heat also involves hydrogen sulfide and JA, the synthesis of which is increased under high-temperature stress [47]. Here, JA/H_2_S signaling controlled the high-temperature induction of nicotine synthesis. Suppression of H_2_S signaling using its scavengers and synthesis inhibitors or inhibition of L-cysteine desulfhydrase gene expression in transgenic plants caused disruption of JA formation and nicotine biosynthesis under high-temperature exposure. However, these inhibitory effects were reversed by the application of exogenous H_2_S. The authors suggested that H_2_S acts upstream of JA as a signaling molecule under high-temperature stress (Figure 3) and is a trigger for nicotine biosynthesis in tobacco [47]. The role of hydrogen sulfide in inducing JA synthesis has also been indicated by data from bioinformatics methods, according to which almost half of the proteins associated with jasmonate biosynthesis are persulfidated [166].

As mentioned above, one of the key proteins in jasmonate signaling, JIN1/MYC2, may be involved in realizing the effects of JA, as well as other participants in the signaling network such as the gasotransmitter NO. Data have also been obtained, indicating its possible involvement in the stress-protective effect of hydrogen sulfide on plants. Recently, using molecular genetics techniques, the activation of expression of the gene encoding transcription factor MYC2 and the involvement of endogenous hydrogen sulfide as a signal mediator in this process has been shown upon enhancing the adaptation of Arabidopsis plants to hypoxia (flooding) in the dark by the exogenous hydrogen sulfide action [167]. This work also shows the involvement of endogenous hydrogen sulfide as a signaling mediator in the activation of MYC2 gene expression (Figure 3).

Comparison of the effect of the hydrogen sulfide donor NaHS on Arabidopsis wild-type (Col-0) plants and *jin1* mutants showed an increase in salt tolerance of Col-0, which was expressed as a decrease in oxidative damage, a reduction in water deficiency, and preservation of the photosynthetic pigment pool under salt stress [168]. In addition, treatment of wild-type plants with H_2_S donor prevented the stress-induced decrease in the activity of the antioxidant enzymes SOD and CAT and increased the activity of guaiacol peroxidase. At the same time, exposure of jin1 plants to exogenous H_2_S did not increase their salt tolerance or alter the state of their stress-protective systems. These phenomena suggest the involvement of the transcription factor JIN1/MYC2 in the physiological effects of hydrogen sulfide [168]. The mechanisms underlying the effect of hydrogen sulfide signaling on this transcription factor remain unclear. It is not excluded that it is mediated and associated with changes in other signaling pathways and possibly hormonal mediators under the influence of H_2_S. In particular, the protective effects of hydrogen sulfide may also be mediated by its effect on the content of various plant stress hormones, such as ABA [169], whose signal transduction also involves the JIN1/MYC2 protein [130]. There is evidence that a key transcription factor in jasmonate signaling is involved in the effects of other plant hormones and signaling compounds, including the gasotransmitter H_2_S.

At the same time, hydrogen sulfide may mediate the physiological effects of JA, particularly its influence on redox homeostasis, with the involvement of MAP kinases in this process [170] (Figure 3). Treatment of wild-type Arabidopsis plants with JA has been shown to significantly increase mitogen-activated protein kinase kinase (MEK1/2) phosphorylation, enhance hydrogen sulfide synthesis, and increase the ascorbate (AsA) to dehydroascorbate (DHA) ratio (AsA/DHA) [171]. However, in the cysteine desulfhydrase gene mutant (*Atl-cdes*), no detectable effect of JA on these processes was observed. The H_2_S scavenger hypotaurine was also found to significantly reduce JA-induced MEK1/2 phosphorylation and AsA/DHA ratio in wild-type plants. However, the authors do not exclude that the effect of hydrogen sulfide on MEK1/2 protein phosphorylation may be more complex and dependent on the functional interaction of hydrogen sulfide with NO and H_2_O_2_ [171]. As already noted, there is experimental evidence indicating their involvement in the regulation of both MEK1/2 and glutathione and ascorbate metabolism (see Section 5 and Section 6.1). Hydrogen sulfide has a very complex interaction with ROS and nitric oxide, with one mechanism being competition for the thiol groups of proteins during their post-translational modification [27]. A discussion of possible models of such interactions in the implementation of JA effects requires a special analysis of a large body of data and is beyond the scope of this review.

The functional interaction between JA and hydrogen sulfide is important for stomatal regulation under drought and other stressors. Thus, the role of hydrogen sulfide in stomatal closure induced by JA action on *Vicia faba* leaves has been demonstrated by the inhibitor method [172]. The effect of JA on the state of guard cells was accompanied by an increase in endogenous hydrogen sulfide and was eliminated by inhibitors of its synthesis, like pyruvate, hydroxylamine, and other compounds. As already noted, the effect of JA on stomata requires the involvement of hydrogen peroxide as one of the mediators of JA signaling. Hydrogen peroxide can influence the synthesis of hydrogen sulfide and stomatal closure depends on the functional interactions of these molecules [172].

JA and hydrogen sulfide are not only involved in the regulation of stomatal aperture size but also in the development of the stomatal apparatus. It has been shown that JA-deficient Arabidopsis mutants were characterized by high stomatal density. However, this effect was eliminated by the exogenous H_2_S treatment. In contrast, *lcd* mutants deficient in H_2_S synthesis had a stomata development phenotype similar to that of JA-deficient mutants. This effect was not observed when plants were treated with a hydrogen sulfide donor, but not with JA [173]. Thus, H_2_S can act as a downstream signalling component that alters stomatal development.

### 6.3. Carbon Monoxide

CO, as a gasotransmitter in plant cells, is still less well studied than NO and H_2_S. There is evidence that CO is involved in seed germination, adventitious rooting [174], organ aging, and the regulation of many processes related to plant adaptation to adverse factors [82,174,175,176].

Similar to animals, the main source of endogenous CO production in plants is heme oxygenase (HO), which catalyzes the breakdown of heme to form Fe^2+^, biliverdin IXα, and CO [177,178]. Plant heme oxygenases are represented by a family of four genes, of which *HO1* is the most intensively expressed [179]. Expression of this gene also increases under stressful conditions [82,180,181].

Unlike other important gasotransmitters (NO and H_2_S), carbon monoxide has no redox activity and does not interact with the thiol groups in proteins. Its primary effects may be associated with coordination bonds with metals located in the active centers of the enzymes [82,182].

**Figure 4:**
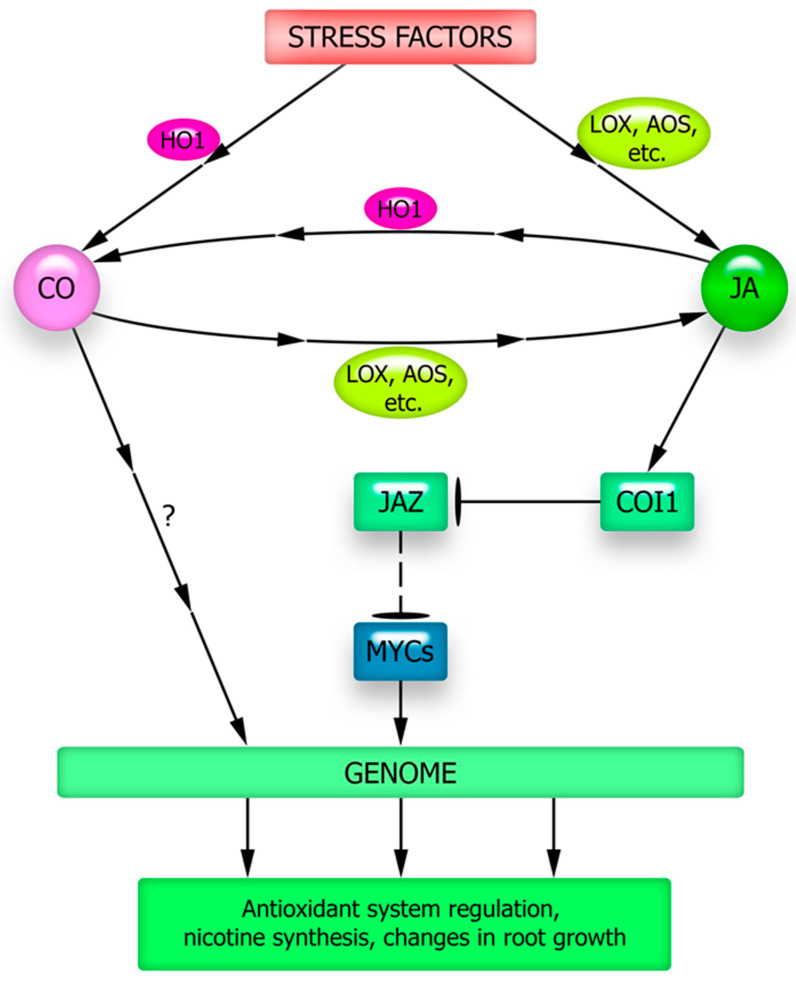
Possible ways of functional interaction of JA and CO in the formation of plant adaptive responses. AOS—allene oxide synthase; COI1—CORONATINE INSENSITIVE1 (ubiquitin ligase complex involved in protein degradation in the 26S proteasome); HO1—heme oxygenase 1; JAZ—JASMONATE ZIM DOMAIN repressor protein; LOX—lipoxygenase; MYCs—transcriptional factors of the MYC family. See the explanations in the text.

There is still insufficient data on the functional relationships between carbon monoxide and JA. However, the involvement of the jasmonate signaling pathway in the CO-mediated induction of nicotine synthesis in tobacco plants exposed to high temperatures has been demonstrated experimentally [183]. Nicotine biosynthesis is limited by the activity of putrescine N-methyltransferase, which methylates putrescine, which is the first step in the nicotine synthesis pathway. Activation of gene expression of this enzyme by NtMYC2a is one result of the heat-induced increase in CO and JA synthesis in tobacco plants. Under normal conditions, NtMYC2a was inhibited by the NtJAZ1 protein. However, the CO-induced increase in JA levels leads to the degradation of NtJAZ1, which releases NtMYC2a and allows it to bind to the NtPMT1 promoter, activating its expression and increasing nicotine biosynthesis [183]. Notably, carbon monoxide donors in tobacco plants increase JA and nicotine synthesis [183]. Thus, there is reason to believe that CO may be an inducer of the JA signaling pathway, which activates nicotine synthesis (Figure 4).

Evidence has also been obtained regarding the role of jasmonate signaling in carbon monoxide-induced protective responses of Arabidopsis plants to salt stress [184]. Arabidopsis plants of the wild-type and jasmonate signaling mutants, *coi1* and *jin1*, were used in this study. In response to treatment with the carbon monoxide donor hemin, only wild-type plants mitigated the development of a water deficit, reduced the degradation of photosynthetic pigments, stabilized the activity of antioxidant enzymes, and increased the accumulation of compatible osmolytes under salt stress. At the same time, *coi1* and *jin1* mutants did not exhibit any of these effects under the influence of the CO donor. Thus, the results obtained indicate the involvement of components of jasmonate signaling in the stress-protective effects of exogenous carbon monoxide [184] (Figure 4). To elucidate the mechanisms underlying the involvement of jasmonate signaling proteins in the implementation of the described CO effects, special studies are needed. It is possible that the role of COI1 and JIN1/MYC2 proteins in the action of exogenous CO is due to the ability of this gasotransmitter to induce JA synthesis.

However, the possible involvement of carbon monoxide as a mediator of JA effects has also been reported (Figure 4). Treatment of soybean plants with JA increased their resistance to Cd, thereby mitigating oxidative damage. This effect of JA is accompanied by an increase in heme oxygenase activity, but not *HO1* gene expression [22]. The authors suggested that JA causes post-translational modifications in heme oxygenase. On the other hand, the effect of MeJA on lateral root formation in rice has been shown to be associated not only with an increase in heme oxygenase activity but also with an increase in *OsHO1* transcripts [185].

## 7. Conclusions and Future Perspectives

JA is one of the key plant hormones that regulate the adaptive responses of plants to biotic and abiotic stressors. This is evidenced by increased JA synthesis when plants are exposed to abiotic stresses; increased plant resistance to stress temperatures, drought, salinity, heavy metals, and other adverse factors under the influence of exogenous JA and its derivatives; reduced resistance to abiotic stress in plants genetically defective in JA synthesis or jasmonate signaling.

JA has a close functional relationship with many components of the signaling network. Signal mediators may be involved both in the induction of JA synthesis and in the transduction of its signals into the genetic apparatus [65,123,186,187]. A number of experiments using various objects and stressors have shown the involvement of the most universal signaling mediators, calcium, and ROS, in the stress-induced activation of JA synthesis. However, the molecular mechanisms of calcium and ROS involvement in JA signaling remain poorly understood. For example, the role of the calcium-regulated JAV1-JAZ8-WRKY51 complex in JA synthesis in response to plant damage by phytophages has been characterized [65], but it is unclear whether this complex is involved in a similar process under other stresses. Many phenomena related to the strengthening and weakening of ROS generation in plant cells under JA action also remain unexplained.

The elucidation of JA’s role in the signaling and hormonal network activated under stress is also complicated by the participation of “canonical” proteins of jasmonate signaling in the effects of other plant hormones and signaling mediators. Thus, the most important transcription factor MYC2, which ensures the realization of the regulatory effects of JA, may be involved in the implementation of the effects of ABA and signal mediators-gasotransmitters (NO, H_2_S, and CO) [100,130,131,132]. Such effects of functional interaction between JA and signaling mediators are necessary for the formation of many plant defense responses to abiotic stressors, such as activation of the antioxidant system, synthesis of osmolytes, formation of stress proteins, regulation of stomata status, and development of stomatal apparatus (Figure 2, Figure 3 and Figure 4).

**Figure 5:**
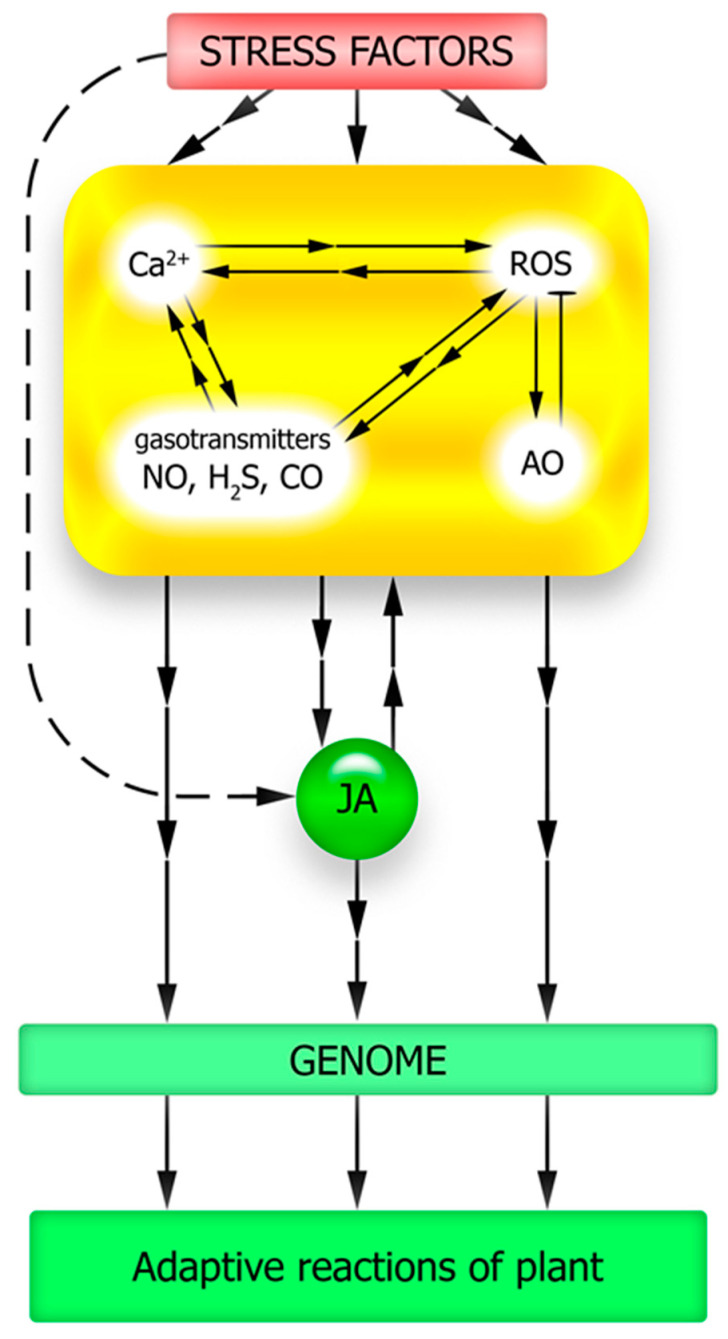
Involvement of key cellular signaling mediators in jasmonate-induced development of plant resistance to abiotic stressors. AO—antioxidant system; ROS—reactive oxygen species.

It is largely unclear how an increase in key signaling mediators (ROS, nitric oxide, and hydrogen sulfide) occurs in cells under JA influence (Figure 5). In other words, the question remains open as to how the currently well-studied set of protein intermediaries that ensure JA signal transduction into the genetic machinery (JAR1, COI1, MYC2) interacts with the universal components of the signaling network (calcium, ROS, and gasotransmitters). Moreover, the complex of signaling mediators that are functionally interacting with JA should presumably be supplemented by metabolites with antioxidant properties. This is indicated by recent data on the possibility of JA synthesis induction under stress conditions in rice plants when treated with proline [105] and in Arabidopsis plants with increased expression of different flavonoid synthase forms and accumulating significantly more flavonoids compared to the wild-type plants [103]. The mechanisms of the antioxidant effects and their possible specificity for the induction of JA synthesis in plants remain to be elucidated. It remains unclear whether the described effects are due to the specific action of flavonoids or proline on JA synthesis and signal transduction processes, or whether they result from changes in the overall cellular redox status. It also remains unclear how the induction of JA synthesis by pro- and antioxidants, as well as the probable involvement of ROS in JA signal transduction, are related to each other. It is reasonable to expect that focusing the researchers’ attention on the effects of functional interactions between JA, non-protein signaling mediators, and stress metabolites will create opportunities for a better understanding of the causes of JA protective effects under abiotic stressor action on plants.

A certain potential for effective induction of plant resistance to abiotic stresses by exogenous factors can be observed in the combined action of JA as a hormone and gasotransmitters as signal mediators involved in the realization of its effects. For example, few data available to date indicate significant synergies in the stress-protective effects of JA and NO acting together in plants [136,188]. Expanding and deepening such research (especially regarding the combined effect of JA and hydrogen sulfide and JA and carbon monoxide) may contribute to the development of new tools for managing plant resistance to a variety of stressors.

## Data Availability

Not applicable.

## References

[1] Hasanuzzaman M., Nahar K., Fujita M., Ahmad P., Azooz M.M., Prasad M.N.V. (2013). Plant response to salt stress and role of exogenous protectants to mitigate salt-induced damages. Ecophysiology and Responses of Plants under Salt Stress.

[2] Raza A., Charagh S., Zahid Z., Mubarik M.S., Javed R., Siddiqui M.H., Hasanuzzaman M. (2021). Jasmonic acid: A key frontier in conferring abiotic stress tolerance in plants. Plant Cell Rep..

[3] Ahmad P., Rasool S., Gul A., Sheikh S.A., Akram N.A., Ashraf M., Kazi A.M., Gucel S. (2016). Jasmonates: Multifunctional roles in stress tolerance. Front. Plant Sci..

[4] Gomi K. (2020). Jasmonic Acid: An essential plant hormone. Int. J. Mol. Sci..

[5] Liu H., Timko M.P. (2021). Jasmonic Acid Signaling and Molecular Crosstalk with Other Phytohormones. Int. J. Mol. Sci..

[6] Hewedy O.A., Elsheery N.I., Karkour A.M., Elhamouly N., Arafa R.A., Mahmoud G.A.-E., Dawood M.F.-A., Hussein W.E., Mansour A., Amin D.H. (2023). Jasmonic acid regulates plant development and orchestrates stress response during tough times. Environ. Exp. Bot..

[7] Jang G., Yoon Y., Choi Y.D. (2020). Crosstalk with jasmonic acid integrates multiple responses in plant development. Int. J. Mol. Sci..

[8] Yang J., Duan G., Li C., Liu L., Han G., Zhang Y., Wang C. (2019). The crosstalks between jasmonic acid and other plant hormone signaling highlight the involvement of jasmonic acid as a core component in plant response to biotic and abiotic stresses. Front. Plant Sci..

[9] Wang X., Li Q., Xie J., Huang M., Cai J., Zhou Q., Dai T., Jiang D. (2021). Abscisic acid and jasmonic acid are involved in drought priming-induced tolerance to drought in wheat. Crop J..

[10] Li T., Feng M., Chi Y., Shi X., Sun Z., Wu Z., Li A., Shi W. (2023). Defensive resistance of cowpea *Vigna unguiculata* control megalurothrips usitatus mediated by jasmonic acid or insect damage. Plants.

[11] Rohwer C.L., Erwin J.E. (2008). Horticultural applications of jasmonates: A review. J. Hortic. Sci. Biotechnol..

[12] Nawaz F., Shabbir R.N., Shahbaz M., Majeed S., Raheel M., Hassan W., Sohail M.A., El-Esawi M.A. (2017). Cross talk between nitric oxide and phytohormones regulate plant development during abiotic stresses. Phytohormones—Signaling Mechanisms and Crosstalk in Plant Development and Stress Responses.

[13] Yu X., Zhang W., Zhang Y., Zhang X., Lang D., Zhang X. (2019). The roles of methyl jasmonate to stress in plants. Funct. Plant Biol..

[14] Sahil A.D., Mehta S., Abdelmotelb K.F., Lavale S.A., Aggarwal S.K., Jat B.S., Tripathi A., Garg S., Husen A. (2021). Jasmonic acid for sustainable plant growth and production under adverse environmental conditions. Plant Performance under Environmental Stress.

[15] Ghorbel M., Brini F., Sharma A., Landi M. (2021). Role of jasmonic acid in plants: The molecular point of view. Plant Cell Rep..

[16] Barrera-Ortiz S., Garnica-Vergara A., Esparza-Reynoso S., García-Cárdenas E., Raya-González J., Ruiz-Herrera L.F., López-Bucio J. (2018). Jasmonic acid-ethylene crosstalk via *ETHYLENE INSENSITIVE* 2 reprograms *Arabidopsis* root system architecture through nitric oxide accumulation. J. Plant Growth Regul..

[17] Ho T.-T., Murthy H.N., Park S.-Y. (2020). Methyl jasmonate induced oxidative stress and accumulation of secondary metabolites in plant cell and organ cultures. Int. J. Mol. Sci..

[18] Lee H.J., Seo P.J. (2021). Ca^2+^-talyzing initial responses to environmental stresses. Trends Plant Sci..

[19] Lu M., Zhang Y., Tang S., Pan J., Yu Y., Han J., Li Y., Du X., Nan Z., Sun Q. (2016). AtCNGC2 is involved in jasmonic acid-induced calcium mobilization. J. Exp. Bot..

[20] Aslam S., Gul N., Mir M.A., Asgher M., Al-Sulami N., Abulfaraj A.A., Qari S. (2021). Role of jasmonates, calcium, and glutathione in plants to combat abiotic stresses through precise signaling cascade. Front. Plant Sci..

[21] Karpets Y.V., Kolupaev Y.E., Lugovaya A.A., Oboznyi A.I. (2014). Effect of jasmonic acid on the pro-/antioxidant system of wheat coleoptiles as related to hyperthermia tolerance. Russ. J. Plant Physiol..

[22] Noriega G., Cruz D.S., Batlle A., Tomaro M., Balestrasse K. (2012). Heme oxygenase is involved in the protection exerted by jasmonic acid against cadmium stress in soybean roots. J. Plant Growth Regul..

[23] Kolupaev Y.E., Karpets Y.V., Beschasniy S.P., Dmitriev A.P. (2019). Gasotransmitters and their role in adaptive reactions of plant cells. Cytol. Genet..

[24] Yao Y., Yang Y., Li C., Huang D., Zhang J., Wang C., Li W., Wang N., Deng Y., Liao W. (2019). Research progress on the functions of gasotransmitters in plant responses to abiotic stresses. Plants.

[25] Gotor C., García I., Aroca Á., Laureano-Marín A.M., Arenas-Alfonseca L., Jurado-Flores A., Moreno I., Romero L.C. (2019). Signaling by hydrogen sulfide and cyanide through post-translational modification. J. Exp. Bot..

[26] Shivaraj S.M., Vats S., Bhat J.A., Dhakte P., Goyal V., Khatri P., Kumawat S., Singh A., Prasad M., Sonah H. (2020). Nitric oxide and hydrogen sulfide crosstalk during heavy metal stress in plants. Physiol. Plant..

[27] Kolupaev Y.E., Yemets A.I., Yastreb T.O., Blume Y.B. (2023). The role of nitric oxide and hydrogen sulfide in regulation of redox homeostasis at extreme temperatures in plants. Front. Plant Sci..

[28] Wasternack C., Strnad M. (2018). Jasmonates: News on occurrence, biosynthesis, metabolism and action of an ancient group of signaling compounds. Int. J. Mol. Sci..

[29] Wasternack C., Hause B. (2013). Jasmonates: Biosynthesis, perception, signal transduction and action in plant stress response, growth and development. An update to the 2007 review in Annals of Botany. Ann. Bot..

[30] Wang F., Yu G., Liu P. (2019). Transporter-mediated subcellular distribution in the metabolism and signaling of jasmonates. Front. Plant Sci..

[31] Santino A., Taurino M., De Domenico S., Bonsegna S., Poltronieri P., Pastor V., Flors V. (2013). Jasmonate signaling in plant development and defense response to multiple (a)biotic stresses. Plant Cell Rep..

[32] Ali M.S., Baek K.H. (2020). Jasmonic acid signaling pathway in response to abiotic stresses in plants. Int. J. Mol. Sci..

[33] Li J., Zhang K., Meng Y., Hu J., Ding M., Bian J., Yan M., Han J., Zhou M. (2018). Jasmonic acid/ethylene signaling coordinates hydroxycinnamic acid amides biosynthesis through ORA59 transcription factor. Plant J..

[34] Fonseca S., Chini A., Hamberg M., Adie B., Porzel A., Kramell R., Miersch O., Wasternack C., Solano R. (2009). (+)-7-iso-Jasmonoyl-L-isoleucine is the endogenous bioactive jasmonate. Nat. Chem. Biol..

[35] Ruan J., Zhou Y., Zhou M., Yan J., Khurshid M., Weng W., Cheng J., Zhang K. (2019). Jasmonic acid signaling pathway in plants. Int. J. Mol. Sci..

[36] Zhang C., Huang Z. (2013). Effects of endogenous abscisic acid, jasmonic acid, polyamines, and polyamine oxidase activity in tomato seedlings under drought stress. Sci. Hortic..

[37] Kang D., Seo Y., Lee J.D., Ishii R., Kim K.U., Shin D.H., Park S.K., Lee I. (2005). Jasmonic acid differentially affects growth, ion uptake and abscisic acid concentration in salt-tolerant and salt-sensitive rice cultivars. J. Agron. Crop Sci..

[38] Ryu H., Cho Y.G. (2015). Plant hormones in salt stress tolerance. J. Plant Biol..

[39] Prerostova S., Dobrev P.I., Gaudinova A., Hosek P., Soudek P., Knirsch V., Vankova R. (2017). Hormonal dynamics during salt stress responses of salt-sensitive *Arabidopsis thaliana* and salt-tolerant *Thellungiella salsuginea*. Plant Sci..

[40] Walia H., Wilson C., Wahid A., Condamine P., Cui X., Close Timothy J. (2006). Expression analysis of barley (*Hordeum vulgare* L.) during salinity stress. Funct. Integr. Genom..

[41] Ojuederie O.B., Babalola O.O. (2017). Microbial and plant-assisted bioremediation of heavy metal polluted environments: A review. Int. J. Environ. Res. Public Health.

[42] Rakwal R., Tamogami S., Kodama O. (1996). Role of jasmonic acid as a signaling molecule in copper chloride-elicited rice phytoalexin production. Biosci. Biotechnol. Biochem..

[43] Du H., Liu H., Xiong L. (2013). Endogenous auxin and jasmonic acid levels are differentially modulated by abiotic stresses in rice. Front. Plant Sci..

[44] Hu Y., Jiang L., Wang F., Yu D. (2013). Jasmonate regulates the Inducer of CBF expression–C-repeat binding factor/DRE binding factor1 cascade and freezing tolerance in Arabidopsis. Plant Cell.

[45] Liu W., Wang H., Chen Y., Zhu S., Chen M., Lan X., Chen G., Liao Z. (2017). Cold stress improves the production of artemisinin depending on the increase in endogenous jasmonate. Biotechnol. Appl. Biochem..

[46] Xu Y.H., Liao Y.C., Zhang Z., Liu J., Sun P.-W., Gao Z.-H., Sui C., Wei J.-H. (2016). Jasmonic acid is a crucial signal transducer in heat shock induced sesquiterpene formation in *Aquilaria sinensis*. Sci. Rep..

[47] Chen X., Chen Q., Zhang X., Li R., Jia Y., Ef A.A., Jia A., Hu L., Hu X. (2016). Hydrogen sulfide mediates nicotine biosynthesis in tobacco (*Nicotiana tabacum*) under high temperature conditions. Plant Physiol. Biochem..

[48] Li Q., Zheng J., Li S., Huang G., Skilling S.J., Wang L., Li L., Li M., Yuan L., Liu P. (2017). Transporter-mediated nuclear entry of jasmonoyl-isoleucine is essential for jasmonate signaling. Mol. Plant.

[49] Thines B., Katsir L., Melotto M., Niu Y., Mandaokar A., Liu G., Nomura K., He Y.S., Howe G.A., Browse J. (2007). JAZ repressor proteins are targets of the SCF^COI1^ complex during jasmonate signalling. Nature.

[50] Dombrecht B., Xue G.P., Sprague S.J., Kirkegaard J.A., Ross J.J., Reid J.B., Fitt G.P., Sewelam N., Schenk P.M., Manners J.M. (2007). MYC2 differentially modulates diverse jasmonate-dependent functions in Arabidopsis. Plant Cell.

[51] Lorenzo O., Piqueras R., Sanchez-Serrano J.J., Solano R. (2003). Ethylene response factor1 integrates signals from ethylene and jasmonate pathways in plant defense. Plant Cell.

[52] Nuruzzaman M., Sharoni A.M., Kikuchi S. (2013). Roles of NAC transcription factors in the regulation of biotic and abiotic stress responses in plants. Front. Microbiol..

[53] Chen W.-J., Wang X., Yan S., Huang X., Yuan H.-M. (2019). The ICE-like transcription factor HbICE2 is involved in jasmonate-regulated cold tolerance in the rubber tree (*Hevea brasiliensis*). Plant Cell Rep..

[54] Li Y., Liu Y., Jin L., Peng R. (2022). Crosstalk between Ca^2+^ and other regulators assists plants in responding to abiotic stress. Plants.

[55] Gao Y.L., Zhang G.Z. (2019). A calcium sensor calcineurin B-like 9 negatively regulates cold tolerance *via* calcium signaling in *Arabidopsis thaliana*. Plant Signal. Behav..

[56] Kolupaev Y.E., Yastreb T.O., Ryabchun N.I., Yemets A.I., Dmitriev O.P., Blume Y.B. (2023). Cellular mechanisms of the formation of plant adaptive responses to high temperatures. Cytol. Genet..

[57] Zhao Y., Pan Z., Zhang Y., Qu X.L., Zhang Y.G., Yang Y.Q., Jiang X.N., Huang S.J., Yuan M., Schumaker K.S. (2013). The actin-related protein2/3 complex regulates mitochondrial-associated calcium signaling during salt stress in Arabidopsis. Plant Cell.

[58] Bose J., Pottosin I.I., Shabala S.S., Palmgren M.G., Shabala S. (2011). Calcium efflux systems in stress signaling and adaptation in plants. Front. Plant Sci..

[59] Ma X., Li Q.H., Yu Y.N., Qiao Y.M., Haq S.U., Gong Z.H. (2020). The CBL-CIPK pathway in plant response to stress signals. Int. J. Mol. Sci..

[60] Fisahn J., Herde O., Willmitzer L., Peña-Cortés H. (2004). Analysis of the transient increase in cytosolic Ca^2+^ during the action potential of higher plants with high temporal resolution: Requirement of Ca^2+^ transients for induction of jasmonic acid biosynthesis and PINII gene expression. Plant Cell Physiol..

[61] Kim H., Seomun S., Yoon Y., Jang G. (2021). Jasmonic Acid in Plant Abiotic Stress Tolerance and Interaction with Abscisic Acid. Agronomy.

[62] Vadassery J., Reichelt M., Hause B., Gershenzon J., Boland W., Mithöfer A. (2012). CML42-mediated calcium signaling coordinates responses to *Spodoptera herbivory* and abiotic stresses in Arabidopsis. Plant Physiol..

[63] Heyer M., Scholz S.S., Reichelt M., Kunert G., Oelmüller R., Mithöfer A. (2022). The Ca^2+^ sensor proteins CML37 and CML42 antagonistically regulate plant stress responses by altering phytohormone signals. Plant Mol. Biol..

[64] Xia X.J., Zhou Y.H., Shi K., Zhou J., Foyer C.H., Yu J.Q. (2015). Interplay Between Reactive Oxygen Species and Hormones in the Control of Plant Development and Stress Tolerance. J. Exp. Bot..

[65] Yan C., Fan M., Yang M., Zhao J., Zhang W., Su Y., Xiao L., Deng H., Xie D. (2018). Injury Activates Ca^2+^/Calmodulin-Dependent Phosphorylation of JAV1-JAZ8-WRKY51 Complex for Jasmonate Biosynthesis. Mol. Cell.

[66] Karpets Y.V., Kolupaev Y.E., Yastreb T.O., Oboznyi O.I., Shvydenko M.V., Lugova G.A., Vayner A.O. (2013). Reactive oxygen forms and Ca ions as possible intermediaries under the induction of heat resistance of plant cells by jasmonic acid. Ukr. Biochem. J..

[67] Sagi M., Fluhr R. (2001). Superoxide production by plant homologues of the *gp91phox* NADPH oxidase: Modulation of activity by calcium and by tobacco mosaic virus infection. Plant Physiol..

[68] Wong H.L., Pinontoan R., Hayashi K., Tabata R., Yaeno T., Hasegawa K., Kojima C., Yoshioka H., Iba K., Kawasaki T. (2007). Regulation of rice NADPH oxidase by binding of Rac GTPase to its N-terminal extension. Plant Cell.

[69] Siddiqui M.H., Mukherjee S., Alamri S., Ali H.M., Hasan Z., Kalaji H.M. (2022). Calcium and jasmonic acid exhibit synergistic effects in mitigating arsenic stress in tomato seedlings accompanied by antioxidative defense, increased nutrient accumulation and upregulation of glyoxalase system. S. Afr. J. Bot..

[70] Suhita D., Kolla V.A., Vavasseur A., Raghavendra A.S. (2003). Different signaling pathways involved during the suppression of stomatal opening by methyl jasmonate or abscisic acid. Plant Sci..

[71] Shi S., Li S., Asim M., Mao J., Xu D., Ullah Z., Liu G., Wang Q., Liu H. (2018). The Arabidopsis calcium-dependent protein kinases (CDPKs) and their roles in plant growth regulation and abiotic stress responses. Int. J. Mol. Sci..

[72] Popescu S.C., Popescu G.V., Bachan S., Zhang Z., Seay M., Gerstein M., Snyder M., Dinesh-Kumar S.P. (2007). Differential binding of calmodulin-related proteins to their targets revealed through high-density Arabidopsis protein microarrays. Proc. Natl. Acad. Sci. USA.

[73] Yoon H.-K., Kim S.-G., Kim S.-Y., Park C.-M. (2008). Regulation of leaf senescence by NTL9-mediated osmotic stress signaling in Arabidopsis. Mol. Cells.

[74] Gill S.S., Tuteja N. (2010). Reactive oxygen species and antioxidant machinery in abiotic stress tolerance in crop plants. Plant Physiol. Biochem..

[75] Hasanuzzaman M., Bhuyan M.H.M.B., Zulfiqar F., Raza A., Mohsin S.M., Mahmud J.A., Fujita M., Fotopoulos V. (2020). Reactive oxygen species and antioxidant defense in plants under abiotic stress: Revisiting the crucial role of a universal defense regulator. Antioxidants.

[76] Kohli S.K., Khanna K., Bhardwaj R., Abd_Allah E.F., Ahmad P., Corpas F.J. (2019). Assessment of subcellular ROS and NO metabolism in higher plants: Multifunctional signaling molecules. Antioxidants.

[77] Suhita D., Raghavendra A.S., Kwak J.M., Vavasseur A. (2004). Cytoplasmic alkalization precedes reactive oxygen species production during methyl jasmonate’ and abscisic acid-induced stomatal closure. Plant Physiol..

[78] Mika A., Boenisch M.J., Hopff D., Lüthje S. (2010). Membrane-bound guaiacol peroxidases from maize (*Zea mays* L.) roots are regulated by methyl jasmonate, salicylic acid, and pathogen elicitors. J. Exp. Bot..

[79] Skelly M.J., Loake G.J. (2013). Synthesis of redox-active molecules and their signaling functions during the expression of plant disease resistance. Antioxid. Redox Signal..

[80] Kärkönen A., Kuchitsu K. (2015). Reactive oxygen species in cell wall metabolism and development in plants. Phytochemistry.

[81] Minibayeva F., Kolesnikov O., Chasov A., Beckett R.P., Lüthje S., Vylegzhanina N., Buck F., Böttger M. (2009). Wound-induced apoplastic peroxidase activities: Their roles in the production and detoxification of reactive oxygen species. Plant Cell Environ..

[82] Kolupaev Y.E., Karpets Y.V., Shkliarevskyi M.A., Yastreb T.O., Plohovska S.H., Yemets A.I., Blume Y.B. (2022). Gasotransmitters in plants: Mechanisms of participation in adaptive responses. Open Agric. J..

[83] Marcec M.J., Gilroy S., Poovaiah B.W., Tanaka K. (2019). Mutual interplay of Ca^2+^ and ROS signaling in plant immune response. Plant Sci..

[84] Wu J., Ge X. (2004). Oxidative burst, jasmonic acid biosynthesis, and taxol production induced by low-energy ultrasound in *Taxus chinensis* cell suspension cultures. Biotechnol. Bioeng..

[85] Hu X., Wansha L., Chen Q., Yang Y. (2009). Early signals transduction linking the synthesis of jasmonic acid in plant. Plant Signal. Behav..

[86] Surjadinata B.B., Alberto J.-V., Alberto D.D., Jacobo-Velázquez L., Cisneros-Zevallos L. (2021). Physiological role of reactive oxygen species, ethylene, and jasmonic acid on UV light induced phenolic biosynthesis in wounded carrot tissue. Postharvest Biol. Technol..

[87] Torres-Contreras A.M., Nair V., Senés-Guerrero C., Pacheco A., González-Agüero M., Ramos-Parra P.A., Cisneros-Zevallos L., Jacobo-Velázquez D.A. (2023). Cross-Talk and Physiological Role of Jasmonic Acid, Ethylene, and Reactive Oxygen Species in Wound-Induced Phenolic Biosynthesis in Broccoli. Plants.

[88] Maruta T., Inoue T., Tamoi M., Yabuta Y., Yoshimura K., Ishikawa T., Shigeoka S. (2011). Arabidopsis NADPH oxidases, AtrbohD and AtrbohF, are essential for jasmonic acid-induced expression of genes regulated by MYC2 transcription factor. Plant Sci..

[89] Angelini R., Tisi A., Rea G., Chen M.M., Botta M., Federico R., Cona A. (2008). Involvement of polyamine oxidase in wound healing. Plant Physiol..

[90] Yu X.-Z., Chu Y.-P., Zhang H., Lin Y.-J., Tian P. (2021). Jasmonic acid and hydrogen sulfide modulate transcriptional and enzymatic changes of plasma membrane NADPH oxidases (NOXs) and decrease oxidative damage in *Oryza sativa* L. during thiocyanate exposure. Ecotoxicology.

[91] Myers R.J., Fichman Y., Zandalinas S.I., Mittler R. (2023). Jasmonic acid and salicylic acid modulate systemic reactive oxygen species signaling during stress responses. Plant Physiol..

[92] Dai H., Jia G., Shan C. (2015). Jasmonic acid-induced hydrogen peroxide activates MEK1/2 in upregulating the redox states of ascorbate and glutathione in wheat leaves. Acta Physiol. Plant..

[93] Pakar N., Pirasteh-Anosheh H., Emam Y., Pessarakli M. (2016). Barley growth, yield, antioxidant enzymes, and ion accumulation affected by PGRs under salinity stress conditions. J. Plant Nutr..

[94] Sheteiwy M.S., Shao H., Qi W., Daly P., Sharma A., Shaghaleh H., Hamoud Y.A., El-Esawi M.A., Pan R., Wan Q. (2021). Seed priming and foliar application with jasmonic acid enhance salinity stress tolerance of soybean (*Glycine max* L.) seedlings. J. Sci. Food Agric..

[95] Noor J., Ullah A., Saleem M.H., Tariq A., Ullah S., Waheed A., Okla M.K., Al-Hashimi A., Chen Y., Ahmed Z. (2022). Effect of jasmonic acid foliar spray on the morphophysiological mechanism of salt stress tolerance in two soybean varieties (*Glycine max* L.). Plants.

[96] Sofy M.R., Seleiman M.F., Alhammad B.A., Alharbi B.M., Mohamed H.I. (2020). Minimizing adverse effects of Pb on maize plants by combined treatment with jasmonic, salicylic acids and proline. Agronomy.

[97] Sirhindi G., Mir M.A., Abd-Allah E.F., Ahmad P., Gucel S. (2016). Jasmonic acid modulates the physio-biochemical attributes, antioxidant enzyme activity, and gene expression in *Glycine max* under nickel toxicity. Front. Plant Sci..

[98] Farooq M.A., Gill R.A., Islam F., Ali B., Liu H., Xu J., He S., Zhou W. (2016). Methyl jasmonate regulates antioxidant defense and suppresses arsenic uptake in *Brassica napus* L. Front. Plant Sci..

[99] Li L., Lu X., Ma H., Lyu D. (2017). Jasmonic acid regulates the ascorbate–glutathione cycle in *Malus baccata* Borkh. roots under low root-zone temperature. Acta Physiol. Plant..

[100] Yastreb T.O., Kolupaev Y.E., Shvidenko N.V., Dmitriev A.P. (2018). Action of methyl jasmonate and salt stress on antioxidant system of Arabidopsis plants defective in jasmonate signaling genes. Ukr. Biochem. J..

[101] Abouelsaad I., Renault S. (2018). Enhanced Oxidative Stress in the Jasmonic Acid-Deficient Tomato Mutant *def-1* Exposed to NaCl Stress. J. Plant Physiol..

[102] Ahmad R.M., Cheng C., Sheng J., Wang W., Ren H., Aslam M., Yan Y. (2019). Interruption of Jasmonic Acid Biosynthesis Causes Differential Responses in the Roots and Shoots of Maize Seedlings against Salt Stress. Int. J. Mol. Sci..

[103] Zhang M., Lu X., Ren T., Marowa P., Meng C., Wang J., Yang H., Li C., Zhang L., Xu Z. (2023). Heterologous overexpression of *Apocynum venetum* flavonoids synthetase genes improves *Arabidopsis thaliana* salt tolerance by activating the IAA and JA biosynthesis pathways. Front. Plant Sci..

[104] Liang X., Zhang L., Natarajan S.K., Becker D.F. (2013). Proline mechanisms of stress survival. Antioxid. Redox Signal..

[105] Zhang Q., Feng Y.-X., Tian P., Lin Y.-J., Yu X.-Z. (2022). Proline-mediated regulation on jasmonate signals repressed anthocyanin accumulation through the MYB-bHLH-WDR complex in rice under chromium exposure. Front. Plant Sci..

[106] Durner J., Wendehemme D., Klessig D.F. (1998). Defense gene induction in tobacco by nitric oxide, cyclic GMP and cyclic ADP-ribose. Proc. Natl. Acad. Sci. USA.

[107] Peers C., Lefer D.J. (2011). Emerging roles for gasotransmitters. Exp. Physiol..

[108] Wilson I.D., Neill S.J., Hancock J.T. (2008). Nitric oxide synthesis and signaling in plants. Plant Cell Environ..

[109] Corpas F.J., Barroso J.B. (2017). Nitric oxide synthase-like activity in higher plants. Nitric Oxide.

[110] Gupta K.J., Kaiser W.M. (2010). Production and scavenging of nitric oxide by barley root mitochondria. Plant Cell Physiol..

[111] Farnese F.S., Menezes-Silva P.E., Gusman G.S., Oliveira J.A. (2016). When bad guys become good ones: The key role of reactive oxygen species and nitric oxide in the plant responses to abiotic stress. Front. Plant Sci..

[112] Li Q., Lancaster J.R. (2013). Chemical foundations of hydrogen sulfide biology. Nitric Oxide.

[113] Jeandroz S., Wipf D., Stuehr D.J., Lamattina L., Melkonian M., Tian Z., Zhu Y., Carpenter E.J., Wong G.K., Wendehenne D. (2016). Occurrence, structure, and evolution of nitric oxide synthase-like proteins in the plant kingdom. Sci. Signal..

[114] Khan M., Ali S., Al Azzawi T.N.I., Yun B.-W. (2023). Nitric Oxide Acts as a Key Signaling Molecule in Plant Development under Stressful Conditions. Int. J. Mol. Sci..

[115] Guo F.-Q., Okamoto M., Crawford N.M. (2003). Identification of a plant nitric oxide synthase gene involved in hormonal signaling. Science.

[116] Moreau M., Lee G.I., Wang Y., Crane B.R., Klessig D.F. (2008). AtNOS/AtNOA1 is a functional *Arabidopsis thaliana* cGTPase and not a nitric-oxide synthase. J. Biol. Chem..

[117] Karpets Y.V., Kolupaev Y.E., Yastreb T.O., Dmitriev O.P. (2012). Possible pathways of heat resistance induction in plant cells by exogenous nitrogen oxide. Cytol. Genet..

[118] Gross I., Durner J. (2016). In search of enzymes with a role in 3′, 5′-cyclic guanosine monophosphate metabolism in plants. Front. Plant Sci..

[119] Mishra V., Singh P., Tripathi D.K., Corpas F.J., Singh V.P. (2021). Nitric oxide and hydrogen sulfide: An indispensable combination for plant functioning. Trends Plant Sci..

[120] Singh G., Patel A., Tiwari S., Gupta D., Prasad S.M. (2022). Signaling molecules hydrogen sulfide (H_2_S) and nitric oxide (NO): Role in microalgae under adverse environmental conditions. Acta Physiol. Plant..

[121] Karpets Y.V., Kolupaev Y.E., Kosakivska I.V. (2016). Nitric oxide and hydrogen peroxide as signal mediators at induction of heat resistance of wheat plantlets by exogenous jasmonic and salicylic acids. Fiziol. Rast. Genet..

[122] Sami F., Faizan M., Faraz A., Siddiqui H., Yusuf M., Hayat S. (2018). Nitric oxide-mediated integrative alterations in plant metabolism to confer abiotic stress tolerance, NO crosstalk with phytohormones and NO-mediated post translational modifications in modulating diverse plant stress. Nitric Oxide.

[123] Huang X., Stettmaier K., Michel C., Hutzler P., Mueller M.J., Durner J. (2004). Nitric oxide is induced by wounding and influences jasmonic acid signaling in *Arabidopsis thaliana*. Planta.

[124] Wendehenne D., Durner J., Klessig D.F. (2004). Nitric oxide: A new player in plant signalling and defence responses. Curr. Opin. Plant Biol..

[125] Mur L.A.J., Prats E., Pierre S., Hall M.A., Hebelstrup K.H. (2013). Integrating nitric oxide into salicylic acid and jasmonic acid/ethylene plant defense pathways. Front. Plant Sci..

[126] Verma N., Tiwari S., Singh V.P., Prasad S.M. (2020). Nitric oxide in plants: An ancient molecule with new tasks. Plant Growth Regul..

[127] Zhou J., Ran Z.F., Yang X.T., Li J. (2019). Postharvest UV-B irradiation stimulated ginsenoside Rg_1_ biosynthesis through nitric oxide (NO) and jasmonic acid (JA) in *Panax quinquefolius* roots. Molecules.

[128] Xu M.-J., Dong J.-F. (2008). Synergistic action between jasmonic acid and nitric oxide in inducing matrine accumulation of *Sophora flavescens* suspension cells. J. Integr. Plant Biol..

[129] Singh P.K., Indoliya Y., Chauhan A.S., Singh S.P., Singh A.P., Dwivedi S., Tripathi R.D., Chakrabarty D. (2017). Nitric oxide mediated transcriptional modulation enhances plant adaptive responses to arsenic stress. Sci. Rep..

[130] Ton J., Flors V., Mauch-Mani B. (2009). The multifaceted role of ABA in disease resistance. Trends Plant Sci..

[131] Palmieri M.C., Sell S., Huang X., Scherf M., Werner T., Durner J., Lindermayr C. (2008). Nitric oxide-responsive genes and promoters in *Arabidopsis thaliana*: A bioinformatics approach. J. Exp. Bot..

[132] Yastreb T.O., Kolupaev Y.E., Karpets Y.V., Dmitriev A.P. (2017). Effect of nitric oxide donor on salt resistance of Arabidopsis *jin1* mutants and wild-type plants. Russ. J. Plant Physiol..

[133] Zhang P., Wang X., Lu Q., Zhang H., Chen J., Zhang H., Wang Y., Li C. (2023). Allantoin, a purine metabolite, confers saline–alkaline tolerance to sugar beet by triggering a self-amplifying feedback loop comprising jasmonic acid and nitric oxide. Environ. Exp. Bot..

[134] Liu Y., Yang X., Zhu S., Wang Y. (2016). Postharvest application of MeJA and NO reduced chilling injury in cucumber (*Cucumis sativus*) through inhibition of H_2_O_2_ accumulation. Postharvest Biol. Technol..

[135] Shan C., Zhang S., Ou X. (2018). The roles of H_2_S and H_2_O_2_ in regulating AsA-GSH cycle in the leaves of wheat seedlings under drought stress. Protoplasma.

[136] Ahmad P., Ahanger M.A., Alyemeni M.N., Wijaya L., Alam P., Ashraf M. (2018). Mitigation of sodium chloride toxicity in *Solanum lycopersicum* L. by supplementation of jasmonic acid and nitric oxide. J. Plant Interact..

[137] Melotto M., Mecey C., Niu Y., Chung H.S., Katsir L., Yao J., Zeng W., Thines B., Staswick P., Browse J. (2008). A critical role of two positively charged amino acids in the Jas motif of Arabidopsis JAZ proteins in mediating coronatine- and jasmonoyl isoleucine-dependent interactions with the COI1 F-box protein. Plant J..

[138] Savchenko T., Kolla V.A., Wang C.Q., Nasafi Z., Hicks D.R., Phadungchob B., Chehab W.E., Brandizzi F., Froehlich J., Dehesh K. (2014). Functional convergence of oxylipin and abscisic acid pathways controls stomatal closure in response to drought. Plant Physiol..

[139] Liu X., Shi W., Zhang S., Lou C. (2005). Nitric oxide involved in signal transduction of jasmonic acid-induced stomatal closure of *Vicia faba* L. Chin. Sci. Bull..

[140] Munemasa S., Oda K., Watanabe-Sugimoto M., Nakamura Y., Shimoishi Y., Murata Y. (2007). The *coronatine-insensitive 1* mutation reveals the hormonal signaling interaction between abscisic acid and methyl jasmonate in Arabidopsis guard cells. Specific impairment of ion channel activation and second messenger production. Plant Physiol..

[141] Munemasa S., Mori I.C., Murata Y. (2011). Methyl jasmonate signaling and signal crosstalk between methyl jasmonate and abscisic acid in guard cells. Plant Signal. Behav..

[142] Nazareno A.L., Hernandez B.S. (2017). A mathematical model of the interaction of abscisic acid, ethylene and methyl jasmonate on stomatal closure in plants. PLoS ONE.

[143] Yastreb T.O., Kolupaev Y.E., Kokorev A., Horielova E.I., Dmitriev A.P. (2018). Methyl jasmonate and nitric oxide in regulation of the stomatal apparatus of *Arabidopsis thaliana*. Cytol. Genet..

[144] Zhang H., Hu S.L., Zhang Z.J., Hu L.Y., Jiang C.X., Wei Z.J., Liu J., Wang H.L., Jiang S.T. (2011). Hydrogen sulfide acts as a regulator of flower senescence in plants. Postharvest Biol. Technol..

[145] Li Z.G., Gong M., Liu P. (2012). Hydrogen sulfide is a mediator in H_2_O_2_-induced seed germination in *Jatropha curcas*. Acta Physiol. Plant..

[146] Ziogas V., Molassiotis A., Fotopoulos V., Tanou G. (2018). Hydrogen sulfide: A potent tool in postharvest fruit biology and possible mechanism of action. Front. Plant Sci..

[147] Pandey A.K., Gautam A. (2020). Stress responsive gene regulation in relation to hydrogen sulfide in plants under abiotic stress. Physiol. Plant..

[148] Romero L.C., García I., Gotor C. (2013). L-cysteine desulfhydrase 1 modulates the generation of the signaling molecule sulfide in plant cytosol. Plant Signal. Behav..

[149] Yuan S., Shen X., Kevil C.G. (2017). Beyond a gasotransmitter: Hydrogen sulfide and polysulfide in cardiovascular health and immune response. Antioxid. Redox Signal..

[150] Filipovic M.R., Zivanovic J., Alvarez B., Banerjee R. (2018). Chemical biology of H_2_S signaling through persulfidation. Chem. Rev..

[151] Paul B.D., Snyder S.H. (2018). Gasotransmitter hydrogen sulfide signaling in neuronal health and disease. Biochem. Pharmacol..

[152] Aroca A., Zhang J., Xie Y., Romero L.C., Gotor C. (2021). Hydrogen sulfide signaling in plant adaptations to adverse conditions: Molecular mechanisms. J. Exp. Bot..

[153] Cuevasanta E., Lange M., Bonanata J., Coitiño E.L., Ferrer-Sueta G., Filipovic M.R., Alvarez B. (2015). Reaction of hydrogen sulfide with disulfide and sulfenic acid to form the strongly nucleophilic persulfide. J. Biol. Chem..

[154] Sen N., Paul B.D., Gadalla M.M., Mustafa A.K., Sen T., Xu R., Kim S., Snyder S.H. (2012). Hydrogen sulfide-linked sulfhydration of NF-kB mediates its antiapoptotic actions. Mol. Cell.

[155] Aroca A., Benito J.M., Gotor C., Romero L.C. (2017). Persulfidation proteome reveals the regulation of protein function by hydrogen sulfide in diverse biological processes in Arabidopsis. J. Exp. Bot..

[156] Guo Z., Liang Y., Yan J., Yang E., Li K., Xu H. (2018). Physiological response and transcription profiling analysis reveals the role of H2S in alleviating excess nitrate stress tolerance in tomato roots. Plant Physiol. Biochem..

[157] Kolupaev Y.E., Firsova E.N., Yastreb T.O., Lugovaya A.A. (2017). The participation of calcium ions and reactive oxygen species in the induction of antioxidant enzymes and heat resistance in plant cells by hydrogen sulfide donor. Appl. Biochem. Microbiol..

[158] Fang H., Liu Z., Long Y., Liang Y., Jin Z., Zhang L., Liu D., Li H., Zhai J., Pei Y. (2017). The Ca^2+^/calmodulin2-binding transcription factor TGA3 elevates LCD expression and H_2_S production to bolster Cr^6+^ tolerance in Arabidopsis. Plant J..

[159] Valivand M., Amooaghaie R., Ahadi A. (2019). Interplay between hydrogen sulfide and calcium/calmodulin enhances systemic acquired acclimation and antioxidative defense against nickel toxicity in zucchini. Environ. Exp. Bot..

[160] Wang Y., Li L., Cui W., Xu S., Shen W., Wang R. (2012). Hydrogen sulfide enhances alfalfa (*Medicago sativa*) tolerance against salinity during seed germination by nitric oxide pathway. Plant Soil.

[161] Li Z.G., Yang S.Z., Long W.B., Yang G.X., Shen Z.Z. (2013). Hydrogen sulfide may be a novel downstream signal molecule in nitric oxide-induced heat tolerance of maize (*Zea mays* L.) seedlings. Plant Cell Environ..

[162] Karpets Y.V., Kolupaev Y.E., Lugovaya A.A., Shvidenko N.V., Shkliarevskyi M.A., Yastreb T.O. (2020). Functional interaction of ROS and nitric oxide during induction of heat resistance of wheat seedlings by hydrogen sulfide donor. Russ. J. Plant Physiol..

[163] Sun Y., Qiu X., Ye X., Li Z. (2020). Crosstalk between hydrogen sulfide and nitric oxide signaling in plants. Biotechnol. Bull..

[164] Tian B., Zhang Y., Jin Z., Liu Z., Pei Y. (2017). Role of hydrogen sulfide in the methyl jasmonate response to cadmium stress in foxtail millet. Front. Biosci. Landmark.

[165] Yildirim E., Ekinci M., Turan M., Ors S., Dursun A. (2023). Physiological, Morphological and Biochemical Responses of Exogenous Hydrogen Sulfide in Salt-Stressed Tomato Seedlings. Sustainability.

[166] Filipovic M.R., Jovanovic V.M. (2017). More than just an intermediate: Hydrogen sulfide signalling in plants. J. Exp. Bot..

[167] Yang T., Yuan G., Zhang Q., Xuan L., Li J., Zhou L., Shi H., Wang X., Wang C. (2021). Transcriptome and metabolome analyses reveal the pivotal role of hydrogen sulfide in promoting submergence tolerance in Arabidopsis. Environ. Exp. Bot..

[168] Yastreb T.O., Kolupaev Y.E., Havva E.N., Horielova E.I., Dmitriev A.P. (2020). Involvement of the JIN1/MYC2 transcription factor in inducing salt resistance in Arabidopsis plants by exogenous hydrogen sulfide. Cytol. Genet..

[169] He M., He C.-Q., Ding N.-Z. (2018). Abiotic stresses: General defenses of land plants and chances for engineering multistress tolerance. Front. Plant Sci..

[170] Khan M.S.S., Islam F., Ye Y., Ashline M., Wang D., Zhao B., Fu Z.Q., Chen J. (2022). The interplay between hydrogen sulfide and phytohormone signaling pathways under challenging environments. Int. J. Mol. Sci..

[171] Shan C., Sun H., Zhou Y., Wang W. (2019). Jasmonic acid-induced hydrogen sulfide activates MEK1/2 in regulating the redox state of ascorbate in *Arabidopsis thaliana* leaves. Plant Signal. Behav..

[172] Hou Z., Liu J., Hou L., Li X., Liu X. (2011). H_2_S May function downstream of H_2_O_2_ in jasmonic acid-induced stomatal closure in *Vicia faba*. Chin. Bull. Bot..

[173] Wang C., Deng Y., Liu Z., Liao W. (2021). Hydrogen Sulfide in Plants: Crosstalk with Other Signal Molecules in Response to Abiotic Stresses. Int. J. Mol. Sci..

[174] Yun F., Huang D., Zhang M., Wang C., Deng Y., Gao R., Hou X., Liu Z., Liao W., Liao W. (2022). Comprehensive transcriptome analysis unravels the crucial genes during adventitious root development induced by carbon monoxide in *Cucumis sativus* L. Mol. Biol. Rep..

[175] Zhang S., Li Q., Mao Y. (2014). Effect of carbon monoxide on active oxygen metabolism of postharvest Jujube. J. Food Technol. Res..

[176] Gahir S., Bharath P., Raghavendra A.S. (2020). The role of gasotransmitters in movement of stomata: Mechanisms of action and importance for plant immunity. Biol. Plant..

[177] Shekhawat G.S., Verma K. (2010). Haem oxygenase (HO): An overlooked enzyme of plant metabolism and defence. J. Exp. Bot..

[178] Fang P., Sun T., Wang Y., Wang Y., Ding Y., Pandey A.K., Zhu C., Xu P. (2021). Plant gasotransmitters: Light molecules interplaying with heavy metals. Rev. Environ. Sci. Biotechnol..

[179] Matsumoto F., Obayashi T., Sasaki-Sekimoto Y., Ohta H., Takamiya K.-I., Masuda T. (2004). Gene expression profiling of the tetrapyrrole metabolic pathway in Arabidopsis with a mini-array system. Plant Physiol..

[180] Han Y., Zhang J., Chen X., Gao Z., Xuan W., Xu S., Ding X., Shen W. (2008). Carbon monoxide alleviates cadmium-induced oxidative damage by modulating glutathione metabolism in the roots of *Medicago sativa*. New Phytol..

[181] Bai X., Chen J., Kong X., Todd C.D., Yang Y., Hu X., Li D.Z. (2012). Carbon monoxide enhances the chilling tolerance of recalcitrant *Baccaurea ramiflora* seeds via nitric oxide-mediated glutathione homeostasis. Free. Radic. Biol. Med..

[182] Feelisch M., Olson K.R. (2013). Embracing sulfide and CO to understand nitric oxide biology. Nitric Oxide.

[183] Cheng T., Hu L., Wang P., Yang X., Peng Y., Lu Y., Chen J., Shi J. (2018). Carbon monoxide potentiates high temperature-induced nicotine biosynthesis in Tobacco. Int. J. Mol. Sci..

[184] Shkliarevskyi M.A., Kolupaev Y.E., Yastreb T.O., Karpets Y.V., Dmitriev A.P. (2021). The effect of CO donor hemin on the antioxidant and osmoprotective systems state in Arabidopsis of a wild-type and mutants defective in jasmonate signaling under salt stress. Ukr. Biochem. J..

[185] Hsu Y.Y., Chao Y.-Y., Kao C.H. (2013). Methyl jasmonate-induced lateral root formation in rice: The role of heme oxygenase and calcium. J. Plant Physiol..

[186] Sun Q.-P., Yu Y.-K., Wan S.-X., Zhao F.-K., Hao Y.-L. (2010). Extracellular and intracellular calcium both involved in the jasmonic acid induced calcium mobilization in *Arabidopsis thaliana*. Agric. Sci. China.

[187] Banerjee A., Tripathi D.K., Roychoudhury A. (2018). Hydrogen sulphide trapeze: Environmental stress amelioration and phytohormone crosstalk. Plant Physiol. Biochem..

[188] Zhou J., Jia F., Shao S., Zhang H., Li G., Xia X., Zhou Y., Yu J., Shi K. (2015). Involvement of nitric oxide in the jasmonate-dependent basal defense against root-knot nematode in tomato plants. Front. Plant Sci..

